# Circularly polarised luminescence (CPL) control of oligopeptide–Eu(iii) hybridized luminophores by interaction with peptide side chains†

**DOI:** 10.1039/c9ra09708b

**Published:** 2020-01-14

**Authors:** Yuki Mimura, Takuya Sato, Yuki Motomura, Hiroki Yoshikawa, Motohiro Shizuma, Mizuki Kitamatsu, Yoshitane Imai

**Affiliations:** Department of Applied Chemistry, Faculty of Science and Engineering, Kindai University 3-4-1 Kowakae Higashi-Osaka Osaka 577-8502 Japan y-imai@apch.kindai.ac.jp kitamatu@apch.kindai.ac.jp; Department of Biochemistry, Osaka Research Institute of Industrial Science and Technology 1-6-50 Morinomiya, Joto-ku Osaka 536-8553 Japan

## Abstract

Chiral oligopeptide-naphthalene/Eu(iii) hybridized luminophores emit strong circularly polarised solution-state luminescence (CPL) from Eu(iii) at 592 and 614 nm (|*g*_CPL_| ≤ 2.1 × 10^−2^). Although the peptide ligands have matching absolute configurations, the CPL sign is controllable by varying the number of naphthalene units and peptide/Eu(iii) coordination ratio.

## Abstract 2

The CPL sign is controllable by varying the number of naphthalene units and peptide/Eu(iii) coordination ratio.

## Graphical abstract

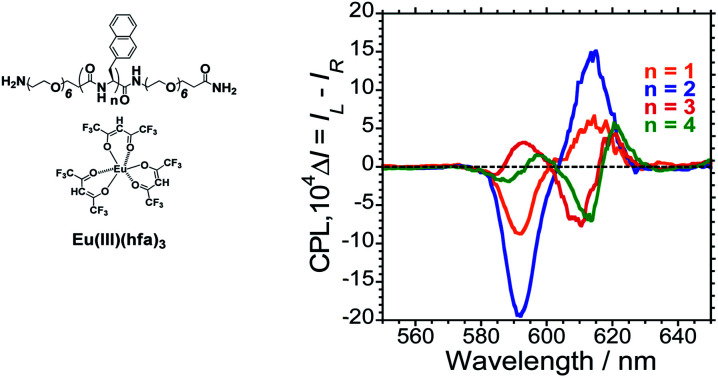

Photoluminescent organic and inorganic materials are used in high-performance electroluminescent and optoelectronic devices. Recently, optically active luminophores that emit circularly polarised luminescence (CPL) with high quantum yield (*Φ*_F_) have received a great deal of interest.^1^ When coordinated by chiral organic ligands, f–f transition metal ions, such as Eu(iii), create strong CPL-emitting luminophores due to high CPL dissymmetry ratio (*g*_CPL_), very narrow full-width at half-maximum (FWHM) bandwidth, and highly tuneable CPL intensity, spanning the visible to near-infrared (NIR) region.^2^

Generally, two enantiomeric organometallic luminophores bearing opposite chirality are required for the selective emission of left-handed and right-handed CPL; however, such chiral organic ligands are not always synthetically tractable. Therefore, it is highly desirable to develop a novel method for controlling the CPL sign of chiral organometallic luminophores without the need to synthesise their respective enantiomers.

Recently, we reported a series of chiral oligopeptide-pyrene luminophores in which the CPL signs of the bipyrenyl-based excimer were successfully controlled by tuning either the distance between the two pyrenyl fluorophores or the solvent.^3^ In addition, the CPL signs of binaphthyl-Eu(iii) hybridized luminophores were successfully inverted by altering the distance between the axial chiral point and the Eu(iii) ion.^4^ Nevertheless, it remains difficult to control the chiroptical properties of metal-based luminophores coordinated to stereoisomeric ligands of the same absolute configuration owing to the metal's multiple coordination sites and the large distance between the metal and the ligand's chiral centre.

Herein, we report oligopeptide–Eu(iii) hybridized luminophores, in which the signs of the Eu(iii) CPL signals at the ^5^D_0_ → ^7^F_1_ and ^5^D_0_ → ^7^F_2_ transitions were subtly inverted in accordance with the number of naphthalene rings in the peptide and the coordination ratio of Eu(iii) to peptide, despite the oligopeptide ligand having the same stereocentres (l or d). Notably, the CPL signals were inverted even though the corresponding chiroptical signs in the circular dichroism (CD) signals of all oligopeptides with l- or d-stereocentres were identical.

Using conventional solid-phase peptide synthesis, we prepared eight compounds comprised of the l- and d-isomers of four chiral oligopeptide–naphthalene organic ligands having different numbers of aromatic naphthalene groups: H-Sp6-l-Ala(Nap)-Sp6-NH_2_ (L-1), H-Sp6-l-Ala(Nap)-L-Ala(Nap)-Sp6-NH_2_ (L-2), H-Sp6-l-Ala(Nap)-l-Ala(Nap)-l-Ala(Nap)-Sp6-NH_2_ (L-3), and H-Sp6-l-Ala(Nap)-l-Ala(Nap)-l-Ala(Nap)-l-Ala(Nap)-Sp6-NH_2_ (L-4), and the corresponding D-isomers, D-1–D-4 (Chart 1; ESI, Fig. S1–S4†). Optically inactive Eu(iii)(hfa)_3_(H_2_O)_2_ [hfa; 1,1,1,5,5,5-hexafluoropentane-1,4-dione] was prepared as previously described (Chart 1).^4^

**Chart 1:**
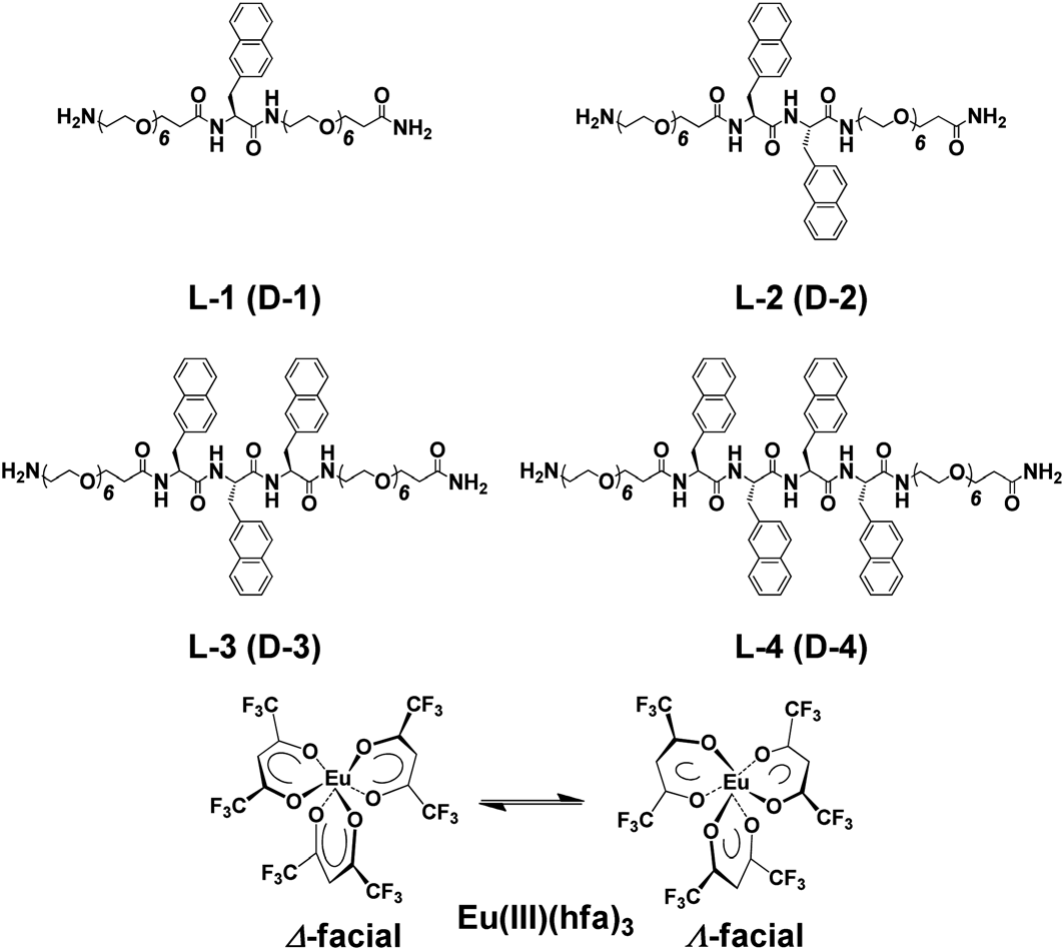
Chiral oligopeptide-naphthalene ligands L-1–L-4 (and D-1–D-4) and Eu(iii)(hfa)_3_.

Four hybridized luminophores, L-1/Eu–L-4/Eu (and D-1/Eu–D-4/Eu), were obtained by mixing the corresponding 1–4 with Eu(iii)(hfa)_3_(H_2_O)_2_ in the desired molar ratio in chloroform (CHCl_3_) at room temperature. We assume that, in the ground state, Eu(iii)(hfa)_3_ exists as a mixture of Δ- and Λ-isomers of *D*_3_-symmetrical facial geometry.

To characterise the photophysical properties of the L-1–L-4/Eu (1/1 molar ratio) luminophores as a function of naphthalene unit number, the steady-state unpolarised photoluminescence (PL) and CPL spectra of dilute L-1–L-4/Eu and L-1–L-4 solutions in CHCl_3_ were obtained (Fig. 1 and ESI, Fig. S5–S9,† and Table 1). Although aggregation-induced quenching regularly limits the use of luminophores, L-1–L-4/Eu remarkably exhibited Eu(iii) PL in CHCl_3_ (Fig. 1, lower panel). The photoluminescence maxima (*λ*_em_) for L-1–L-4/Eu are ∼592 nm (^5^D_0_ → ^7^F_1_), ∼614 nm (^5^D_0_ → ^7^F_2_), ∼651 nm (^5^D_0_ → ^7^F_3_), and ∼698 nm (^5^D_0_ → ^7^F_4_). The values of *λ*_em_ for L-1–L-4/Eu subtly differ from one another by approximately 1−2 nm. The *Φ*_F_ values for L-1–L-4/Eu, mainly arising from the electric-dipole-allowed ^5^D_0_ → ^7^F_2_ transitions, are also similar (0.21, 0.27, 0.34 and 0.32, respectively), owing to the ligands' identical peptide backbone unit.

**Fig. 1:**
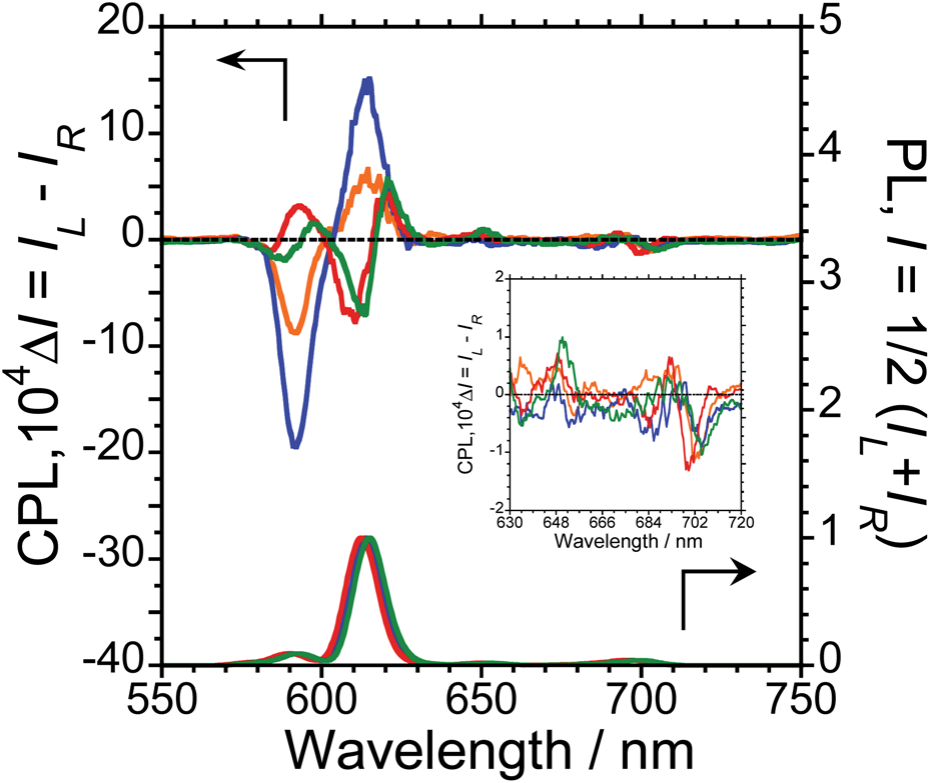
CPL (upper traces) and PL (lower traces) spectra of L-1/Eu (orange lines), L-2/Eu (blue lines), L-3/Eu (red lines), and L-4/Eu (green lines). Conditions: 1.0 × 10^−4^ M L/Eu (1/1 molar ratio) in CHCl_3_, path length = 10 mm, ex: 300 nm. Arrows indicates *y*-axis of respective traces.

**Table 1:** Chiroptical properties of L-1–L-4/Eu (1/1 molar ratio) in CHCl_3_ solution

	CPL				*Φ*_F_	CD	
	*λ*_CPL_ (nm)	*g*_CPL_ (×10^−2^)	*λ*_CPL_ (nm)	*g*_CPL_ (×10^−2^)		*λ*_CD_ (nm)	*g*_CD_ (×10^−4^)
L-1/Eu	592	−0.93	615	+0.067	0.21	330	+3.1
L-2/Eu	592	−2.1	615	+0.15	0.27	329	+5.3
L-3/Eu	593	+0.39	611	−0.083	0.34	333	+4.9
L-4/Eu	597	+0.24	614	−0.074	0.32	334	+0.8

Luminophores L-1–L-4/Eu exhibit clear CPL signals characteristic of ^5^D_0_ → ^7^F_1_ and ^5^D_0_ → ^7^F_2_ transitions (Fig. 1, upper panel). The chiral oligopeptides are thus powerful ligands for Eu(iii)(hfa)_3_ luminescence, affording CPL at 4f–4f transitions. Interestingly, although L-1–L-4 have the same chirality centre, the signs of their CPL spectra for the ^5^D_0_ → ^7^F_1_ and ^5^D_0_ → ^7^F_2_ transitions changed in accordance with the number of naphthalene units in the peptide; specifically, −/+ signs for L-1–L-4/Eu having one or two naphthalene units and −/+/−/+ signs for L-1–L-4/Eu having three or four naphthalene units are clearly observed. As expected for stereoisomers, the CPL spectra of L-1–L-4/Eu and D-1–D-4/Eu almost mirror one another (ESI, Fig. S5b–S8b†). The *g*_CPL_ values in the photoexcited state allow us to quantitatively describe the degree of CPL inducibility provided by L-1–L-4/Eu, defined as *g*_CPL_ = (*I*_L_ – *I*_R_)/[1/2 (*I*_L_ + *I*_R_)] where *I*_L_ and *I*_R_ denote the intensities of the left- and right-handed CPL, respectively, following excitation with unpolarised light. The *g*_CPL_ values for L-1/Eu and L-2/Eu are −0.93 × 10^−2^ at 592 nm and +0.067 × 10^−2^ at 615 nm, and −2.1 × 10^−2^ at 592 nm and +0.15 × 10^−2^ at 615 nm, respectively. The *g*_CPL_ values for L-3/Eu and L-4/Eu are +0.39 × 10^−2^ at 593 nm and −0.083 × 10^−2^ at 611 nm, and +0.24 × 10^−2^ at 597 nm and −0.074 × 10^−2^ at 614 nm, respectively. Overall, L-2/Eu delivers the highest |*g*_CPL_| values among the luminophores. These results suggest that the chirality, l or d, of the ligand's peptide backbone does not determine the CPL signs of Eu(iii) ions at 4f–4f transitions; rather, the CPL signs of peptide ligand-bound Eu(iii) are guided by the number of naphthalene units in the peptide.

We next measured the CD properties of L-1–L-4/Eu (1/1 molar ratio) and L-1–L-4 in CHCl_3_ (Fig. 2 and ESI, Fig. S10–S12,† upper panel, and Table 1). In contrast to the variation observed for the CPL signs, L-1–L-4/Eu complexes commonly exhibit negative (−)-CD spectra at 297 nm, characteristic of the π–π* transitions of the chiral naphthalene units, while the positive (+)-CD spectra at 331 nm arise from the π–π* transitions of both the chiral naphthalene units and the hfa ligands. The CD spectra for L-1–L-4/Eu and D-1–D-4/Eu are mirror images of each other (ESI, Fig. S10†). The dimensionless Kuhn's anisotropy factors for the ground state chirality, *g*_CD_, for 1–4/Eu were ∼+3.1 × 10^−4^ at 330 nm (*λ*_CD_), ∼+5.3 × 10^−4^ at 329 nm (*λ*_CD_), ∼+4.9 × 10^−4^ at 333 nm (*λ*_CD_), and ∼+0.8 × 10^−4^ at 334 nm (*λ*_CD_), respectively.

**Fig. 2:**
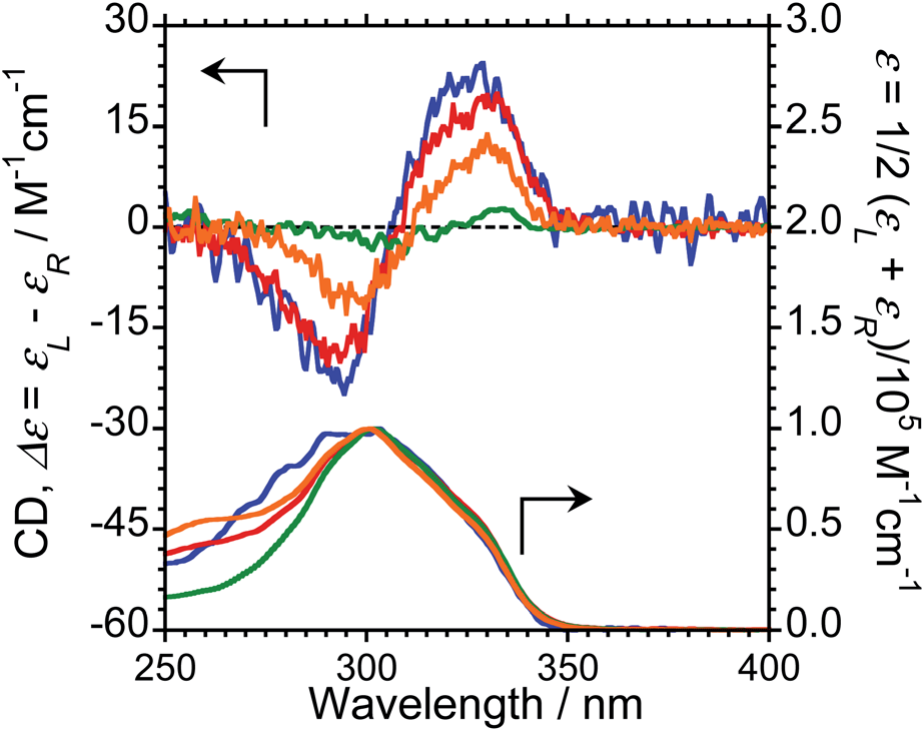
CD (upper traces) and UV-Vis absorption (lower traces) spectra of L-1/Eu (orange lines), L-2/Eu (blue lines), L-3/Eu (red lines), and L-4/Eu (green lines). Conditions: 1.0 × 10^−4^ M L/Eu (1/1 molar ratio) in CHCl_3_, path length = 1 mm. Arrows indicate *y*-axis of respective traces.

Upon comparing the CPL and CD signs of 1–4/Eu, we concluded that the S_0_ ground state chiralities of the luminophores are the same, but the S_1_ photoexcited state chiralities are opposite. In solution, optically inactive Eu(hfa)_3_ exists as a mixture of interconvertible *D*_3_-symmetric Δ- and Λ-isomers, owing to the low barrier height between the two forms. The CPL sign inversion in 1–4/Eu may therefore result from the dynamic switching between the Δ- and Λ-forms of Eu(iii)(hfa)_3_ owing to a dynamic change in the chiral peptide chain, which occurs only in the photo-excited states in the CHCl_3_ solution.

To further expand the CPL control produced by oligopeptide–Eu(iii) hybridized luminophores, we sought to modulate the CPL by varying the peptide/Eu(iii) molar ratio. Concentration-dependent CPL and PL spectra of L-1–L-4/Eu (1/0.5, 1/1, 1/2, 1/3, and 1/4 molar ratios) were obtained using a fixed concentration of [L-2–4] (1.0 × 10^−4^ M in CHCl_3_) (Fig. 3; ESI, Fig. S6–S8†). The CPL intensities at ∼592 nm and ∼614 nm are similar for (L-2/Eu) ratios of 1/0.5 and 1/1. However, when the molar ratio of L-2 to Eu varies from 1/1 to 1/4, these intensities decreased. These results suggest that L-2 coordinates to Eu(iii) in a 1/1 ratio in CHCl_3_. Unfortunately, despite the intensity changes, the CPL sign remained constant.

**Fig. 3:**
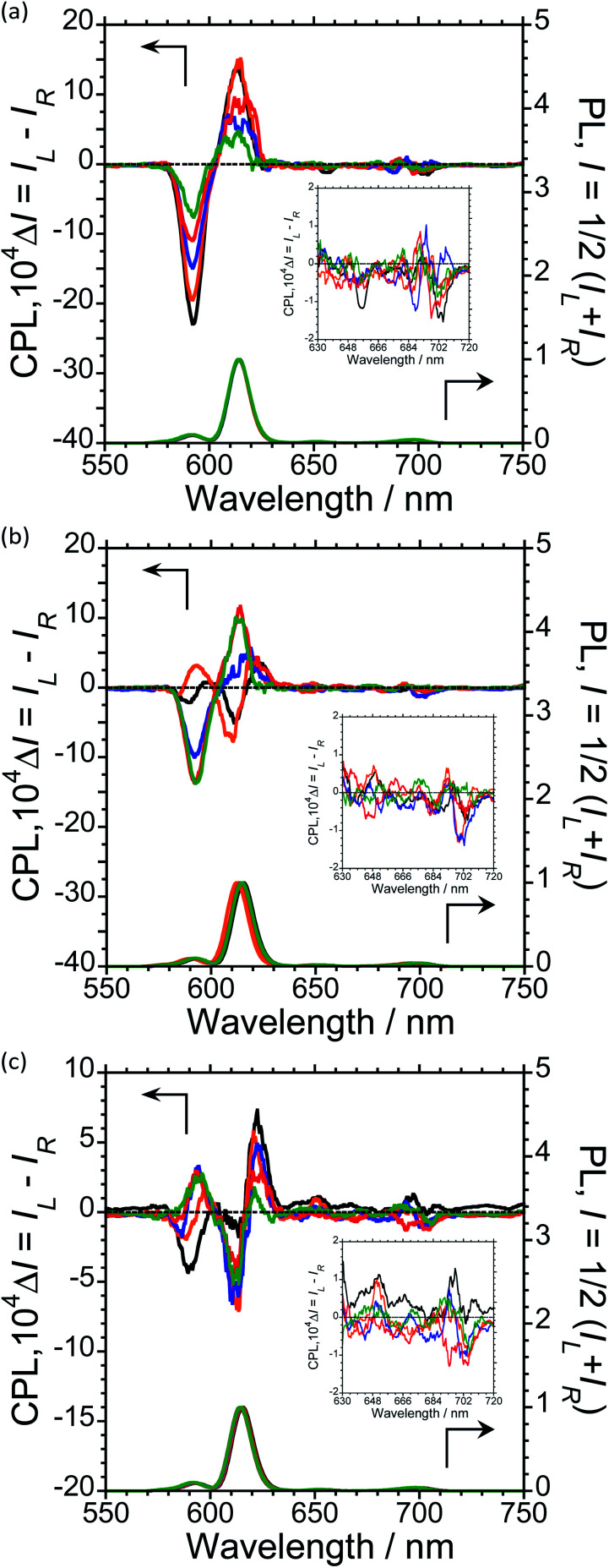
CPL (upper trace) and PL (lower trace) spectra excited at 300 nm of (a) L-2/Eu, (b) L-3/Eu, and (c) L-4/Eu as a function of the molar ratio of Eu to L-2, L-3, and L-4 [L-2–4/Eu = 1/0.5 for black lines, = 1/1 for orange lines, = 1/2 for blue lines, = 1/3 for red lines, and = 1/4 for green lines] in CHCl_3_. Conditions: [L-2–L-4] = 1.0 × 10^−4^ M, path length = 10 mm, ex: 300 nm. Arrows indicate *y*-axis of respective traces.

The CPL spectra of L-3/Eu in CHCl_3_ differ greatly from those of L-2/Eu (Fig. 3(b), ESI, Fig. S7†). Surprisingly, the CPL sign could be controlled by varying the molar ratio of Eu(iii) to L-3. The ^5^D_0_ → ^7^F_1_ and ^5^D_0_ → ^7^F_2_ transitions have decisively opposite CPL signs: −/− signs for 1/0.5 (L-3/Eu) molar ratios, +/− signs for 1/1 (L-3/Eu) molar ratios and −/+ signs for 1/2–4 (L-3/Eu) molar ratios. Additionally, 1/2–4 (L-3/Eu) molar ratios display similar CPL intensities, indicating that L-3 coordinates to Eu(iii) at a maximum 1/2 (L-3/Eu) ratio in CHCl_3_. The CPL sign could also be controlled by varying the molar ratio in the L-4/Eu system (Fig. 3(c), ESI, Fig S8†). While the CPL signs for the ^5^D_0_ → ^7^F_2_ transitions were unchanged, decisively opposite CPL signs for the ^5^D_0_ → ^7^F_1_ transition were observed: − signs for 1/0.5–1 (L-4/Eu) molar ratios and + signs for 1/2–4 (L-4/Eu) molar ratios. These results are consistent with the idea that the CPL sign can be tailored by tuning both the molar ratio of Eu(iii) to peptide and the configurational chirality (l or d) of the oligopeptide.

To investigate the ground-state chirality of different L-3/Eu molar ratios, CD and UV-Vis absorption spectra of 1/0.5–4 L-3/Eu in CHCl_3_ were obtained (Fig. 4; ESI, Fig S10†). Despite the L-3/Eu molar ratio, several characteristic CD bands arising from the π–π* transitions of the naphthalene units and the hfa ligands were observed. There were no CD signal sign changes observed. These results further confirm that there are no differences between the S_0_ ground state chiralities of the four luminophores.

**Fig. 4:**
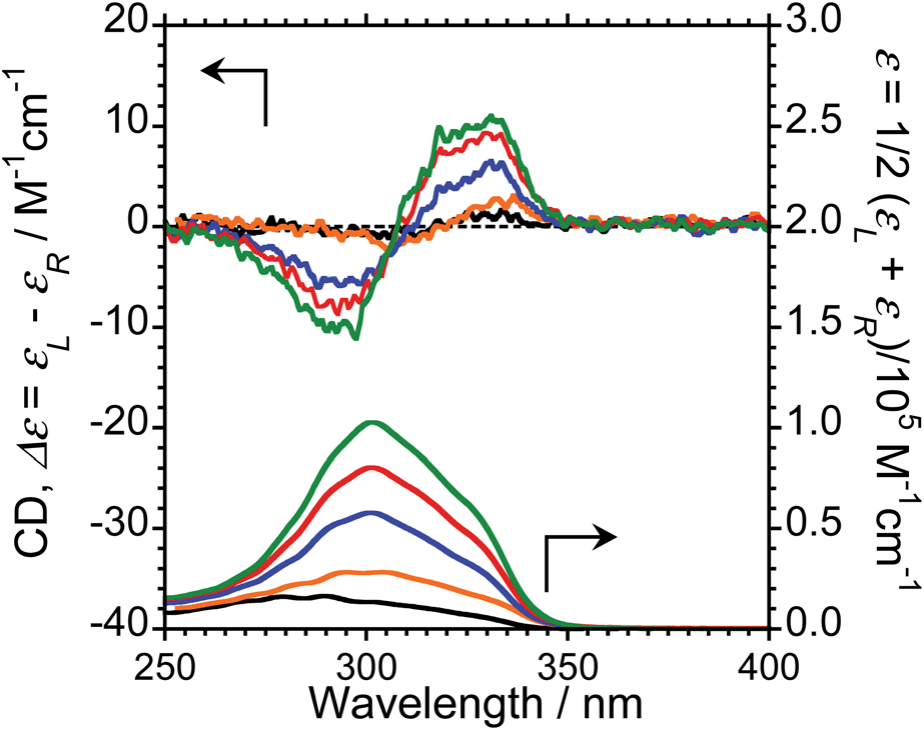
CD (upper trace) and UV-Vis absorption (lower trace) spectra of L-3/Eu as a function of the molar ratio of Eu to L-3 [L-3/Eu = 1/0.5 for black lines, = 1/1 for orange lines, = 1/2 for blue lines, = 1/3 for red lines, and = 1/4 for green lines] in CHCl_3_. Conditions: [L-3] = 1.0 × 10^−4^ M, path length = 1 mm. Arrows indicate *y*-axis of respective traces.

For a fixed concentration of L-2–L-4 (1.0 × 10^−4^ M), the *g*_CPL_ value at the two CPL bands (592 and 614 nm) in CHCl_3_ slightly depended on the molar ratio when changed from 1/0.5 to 1/4 (L-2–4/Eu) (Fig. 5; ESI, Fig. S6–S8†). In L-2, although the |*g*_CPL_| values decreased as the molar ratio of Eu(iii) to L-2 increased from 1/0.5 to 1/4, the slope of the curve drastically changes between L-2/Eu molar ratios of 1/1 and 1/2 (Fig. 5(a); ESI, Fig. S6†). On the other hand, for L-3, the |*g*_CPL_| values increased with an increasing L-3/Eu molar ratio from 1/0.5 to 1/2 (Fig. 5(b); ESI, Fig. S7†). A dramatic slope change was observed between 1/2 and 1/3 (L-3/Eu) molar ratios. Complex changes in the |*g*_CPL_| values were observed for L-4 (Fig. 5(c); ESI, Fig. S8†). These results further confirm that L-2 and L-3 coordinate to Eu(iii) in CHCl_3_ in 1/1 and 1/2 ratios, respectively.

**Fig. 5:**
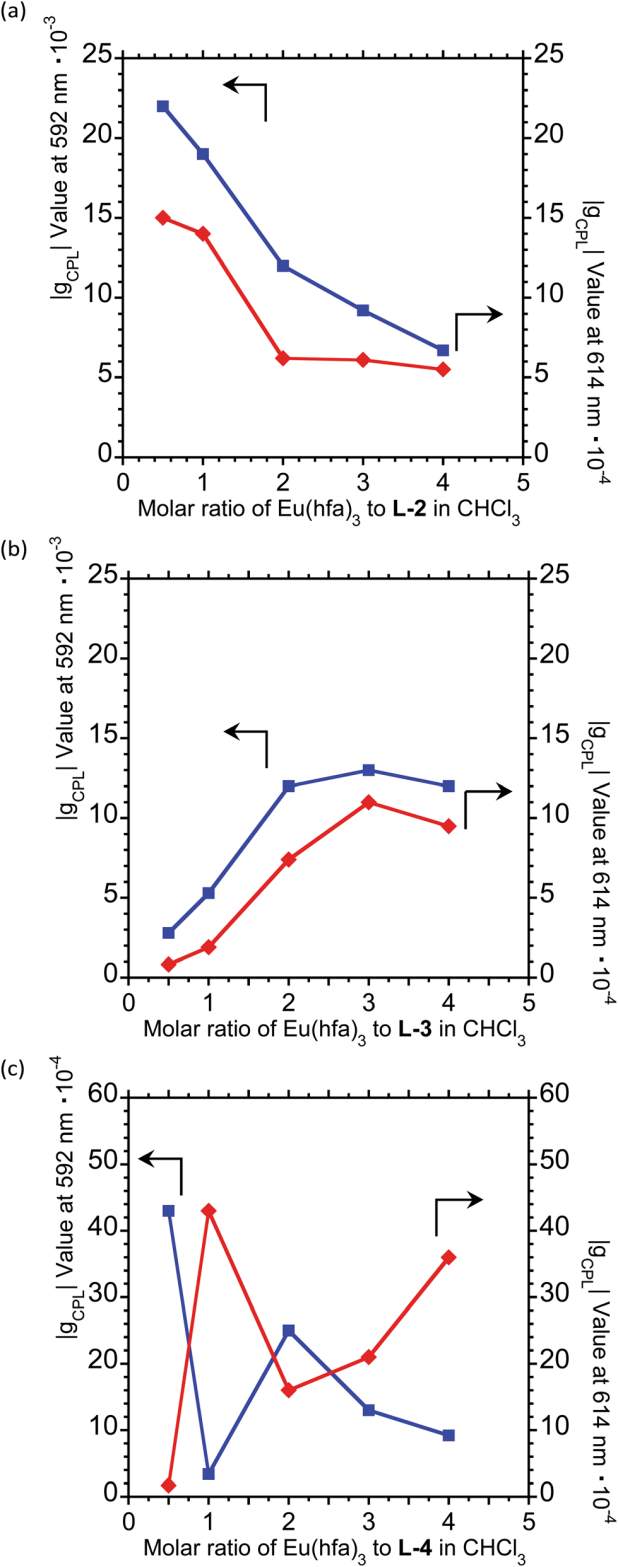
The |*g*_CPL_| values of ^5^D_0_ → ^7^F_1_ (592 nm) (blue lines) and ^5^D_0_ → ^7^F_2_ (614 nm) (red lines) transitions as a function of the molar ratio of Eu to (a) L-2, (b) L-3, and (c) L-4 in CHCl_3_. [L-2–L-4] = 1.0 × 10^−4^ M.

The coordination capacity of the amide units of 1–4 to Eu(iii) was supported by positive mode LCMS-IT-TOF analysis (Table 2 and ESI, Fig. S13 to S18†). This work confirmed that 1–4 coordinated to Eu(iii): [Eu(L-1)(hfa)_2_]^+^ (*m*/*z* 1451.4020) for L-1, [Eu(L-2)(hfa)_2_]^+^ (*m*/*z* 1648.4832) for L-2, [Eu(L-3)(hfa)_3_HNa]^2+^ (*m*/*z* 1037.2530) for L-3, and [Eu(L-4)(hfa)_2_]^+^ (*m*/*z* 2042.6524) for L-4. Although three hfa ligands were observed for L-3/Eu, only two hfa ligands were observed for L-1, L-2, and L-4 complexes. In addition, the molar ratio of Eu(iii) complex to peptide ligand for L-3 changed from 1/1 to 1/3. Unfortunately, in the case of 1/2 and 1/3 (L-3/Eu) ratios, only the signal corresponding to a 1/1 L-3/Eu ratio was observed; *i.e.*, [Eu(L-3)(hfa)_2_]^+^ (*m*/*z* 1845.5684) for L-3/Eu = 1/2 and [Eu(L-3)(hfa)_2_]^+^ (*m*/*z* 1845.5662) for L-3/Eu = 1/3. The coordination of Eu(iii) to O (from the peptide) is rather weak; therefore, the Eu(iii)–O bonds of the hybrid luminophores may easily dissociate under laser-assisted ionisation energy.

**Table 2:** LCMS-IT-TOF analyses of L-1–L-4/Eu

	*m*/*z*	Found Eu complex
L-1	1451.4020	[Eu(L-1)(hfa)_2_]^+^
L-2	1648.4832	[Eu(L-2)(hfa)_2_]^+^
L-3 (L-3/Eu = 1/1)	1037.2530	[Eu(L-3)(hfa)_3_HNa]^2+^
L-3 (L-3/Eu = 1/2)	1845.5684	[Eu(L-3)(hfa)_2_]^+^
L-3 (L-3/Eu = 1/3)	1845.5662	[Eu(L-3)(hfa)_2_]^+^
L-4	2042.6524	[Eu(L-4)(hfa)_2_]^+^

^19^F-NMR spectra of Eu(iii)(hfa)_3_ with 1–4 (1/1) were measured in CDCl_3_ (ESI, Fig. S19–S24†). The ^19^F-NMR spectra of L-1–L-4/Eu in CDCl_3_ indicates that L-1–L-4/Eu exhibit strong resonance signals at −75.23 ppm (L-1), −79.53 ppm (L-2), −79.46 ppm (L-3), and −79.59 ppm (L-4), respectively, and weak resonance signals at −78.85 ppm and −79.59 ppm (L-1), −75.14 ppm (L-2), −75.08 ppm (L-3), −75.23 ppm and −78.85 ppm (L-4), respectively. These clear chemical shift changes were attributed to the dynamic coordination of 1–4 to Eu(iii) in solution. The eighteen fluorine atoms of Eu(hfa)_3_ are no longer magnetically equivalent due to a lowering of the molecular symmetry from *D*_3_ to *C*_1_ upon interaction with L-1–L-4, as the chirality inducing ligands in the ground state and possibly in the photoexcited states. A significant reorganisation of the three strong hfa ligands along with weaker 1–4 ligands to induce chirality towards Eu(iii) ions might occur in the photoexcited state.

The reason behind the CPL sign inversion has not yet been elucidated. The rotational freedom of the peptide backbone in ligands 1–4 is likely the key to imparting the CPL sign-swapping capability in the photoexcited state, even though 1–4/Eu have the same CD sign in the ground state. It is possible that the twists of the peptide chain, naphthalene units, and hfa ligands in these luminophores are opposite only in the photo-excited states in chloroform.

Chiral 1–4/Eu luminophores were easily prepared by mixing the oligopeptide ligands with Eu(iii)(hfa)_3_ in CHCl_3_. By varying the number of naphthalene units in the ligand and the molar ratio of peptide ligands/Eu(iii), the CPL signs of the luminophores could be controlled. Our findings offer a novel method to control the CPL sign of CPL-functionalised Eu(iii)-based luminophores and have obvious applications to other chiral peptide ligands and, potentially, other lanthanide(iii) ions. In addition, these results provide a deeper understanding of how photoexcited, chiral organometallic luminophores can relax to their ground states with minimum to maximum reorganisation, as revealed by the conservation and breaking of signs between the CD and CPL signals.

## Conflicts of interest

There are no conflicts to declare.

## Supplementary Material

RA-010-C9RA09708B-s001
