# Supplementary material for: Circularly polarised luminescence (CPL) control of oligopeptide–Eu(iii) hybridized luminophores by interaction with peptide side chains
Source: RSC Adv. 2020 Jan 14;10(5):2575–80. doi: 10.1039/c9ra09708b (PMC9048765; doi:10.1039/c9ra09708b)
Supplement: RA-010-C9RA09708B-s001 [file RA-010-C9RA09708B-s001.pdf]

Electronic Supplementary Material (ESI) for RSC Advances.
This journal is © The Royal Society of Chemistry 2020

Electronic Supporting Information

Circularly Polarised Luminescence (CPL) Control of Oligopeptide-Eu(III) Hybridized Luminophores by interaction with peptide side chains

Yuki Mimura,[a] Takuya Sato,[a] Yuki Motomura,[a] Hiroki Yoshikawa,[a] Motohiro Shizuma,[b] Mizuki Kitamatsu*[a] and Yoshitane Imai*[a]

[a] Department of Applied Chemistry, Faculty of Science and Engineering, Kindai University, 3-4-1 Kowakae, Higashi-Osaka, Osaka 577-8502, Japan.
E-mail: (Y.I.) y-imai@apch.kindai.ac.jp. (M.K.) kitamatu@apch.kindai.ac.jp.
[b] Department of Biochemistry, Osaka Research Institute of Industrial Science and Technology, 1-6-50 Morinomiya, Joto-ku, Osaka 536-8553, Japan.

Table of Contents

Experimental Section S3

Fig. S1 Mass spectra of **L**-**1** S4

Fig. S2 Mass spectra of **L**-**2** S4

Fig. S3 Mass spectra of **L**-**3** S5

Fig. S4 Mass spectra of **L**-**4** S5

Fig. S5 CPL and PL spectra as a function of **L**-**1** or **D**-**1** / Eu(hfa)$_3$ ratio in chloroform S8

Fig. S6 CPL and PL spectra as a function of **L**-**2** or **D**-**2** / Eu(hfa)$_3$ ratio in chloroform S9

Fig. S7 CPL and PL spectra as a function of **L**-**3** or **D**-**3** / Eu(hfa)$_3$ ratio in chloroform S10

Fig. S8 CPL and PL spectra as a function of **L**-**4** or **D**-**4** / Eu(hfa)$_3$ ratio in chloroform S11

Fig. S9 CPL and PL spectra of **L**- or **D**-**1**~**4** in chloroform S12

Fig. S10 CD and UV-Vis absorption spectra as a function of **L**-**1~4** or **D**-**1~4** / Eu(hfa)$_3$ ratio in chloroform S13

Fig. S11 CD and UV-Vis absorption spectra as a function of **L**-**3** / Eu(hfa)$_3$ ratio in chloroform S14

Fig. S12 CD and UV-Vis absorption spectra of **L**-**1**~**4** in chloroform S15

Fig. S13 Positive-mode electrospray Mass spectrum of **L**-**1** / Eu(hfa)$_3$ (1/1) S16

Fig. S14 Positive-mode electrospray Mass spectrum of **L**-**2** / Eu(hfa)$_3$ (1/1) S17

Fig. S15 Positive-mode electrospray Mass spectrum of **L**-**3** / Eu(hfa)$_3$ (1/1) S17

Fig. S16 Positive-mode electrospray Mass spectrum of **L**-**3** / Eu(hfa)$_3$ (1/2) S18

Fig. S17 Positive-mode electrospray Mass spectrum of **L**-**3** / Eu(hfa)$_3$ (1/3) S19

Fig. S18 Positive-mode electrospray Mass spectrum of **L**-**4** / Eu(hfa)$_3$ (1/1) S19

Fig. S19 ^{19}F-NMR spectrum of Eu(hfa)$_3$ and **L**-**1** / Eu(hfa)$_3$ (1/1) in $CDCl_3$ S20

Fig. S20 ^{19}F-NMR spectrum of Eu(hfa)$_3$ and **L**-**2** / Eu(hfa)$_3$ (1/1) in $CDCl_3$ S20

Fig. S21 ^{19}F-NMR spectrum of Eu(hfa)$_3$ and **L**-**3** / Eu(hfa)$_3$ (1/1) in $CDCl_3$ S20

Fig. S22 ^{19}F-NMR spectrum of Eu(hfa)$_3$ and **L**-**3** / Eu(hfa)$_3$ (1/2) in $CDCl_3$ S21

Fig. S23 ^{19}F-NMR spectrum of Eu(hfa)$_3$ and **L**-**3** / Eu(hfa)$_3$ (1/3) in $CDCl_3$ S21

Fig. S24 ^{19}F-NMR spectrum of Eu(hfa)$_3$ and **L**-**4** / Eu(hfa)$_3$ (1/1) in $CDCl_3$ S21

Experimental Section

Reagents and chemicals

For spectroscopic measurements, spectroscopic-grade $CHCl_3$ (Dojindo [Kumamoto, Japan], Spectrosol) was used. Eu(III)$(hfa)_3(H_2O)_2$ was prepared according to a previously reported method. [1]

Syntheses of chiral peptide-naphthalene ligands 1-4

Fmoc deprotection was carried out with 20% piperidine in DMF (for 7 min at room temperature). After six times washing with DMF, each amino acid was coupled using HBTU/NMM reagents with reaction time of 40 min per coupling at room temperature. No capping step was carried out. After the *N*-terminal Fmoc group was deprotected, the resin was washed with DCM and was treated with 95% TFA, 2.5% water, 2.5% TIS for 1.5 hours at room temperature. The crude peptides were analysed and purified by reversed phase high-pressure liquid chromatography (RP-HPLC) on C18 column with buffer A. 0.1% TFA in water; buffer B, acetonitrile and monitoring at 280 nm with a gradient of 0-100% B gradient over 20 min. The purified peptides were identified with MALDI-TOF mass spectroscopy (matrix, α-CHCA) (Figures S1-S4) and RP-HPLC.

The yields of peptides were 77% (9.8 mg; **L-1**), 67% (10.4 mg; **L-2**), 63% (11.7 mg; **L-3**), 59% (12.6 mg; **L-4**), 82% (10.4 mg; **D-1**), 69% (10.8 mg; **D-2**), 57% (10.5 mg; **D-3**), and 56% (12.0 mg; **D-4**).

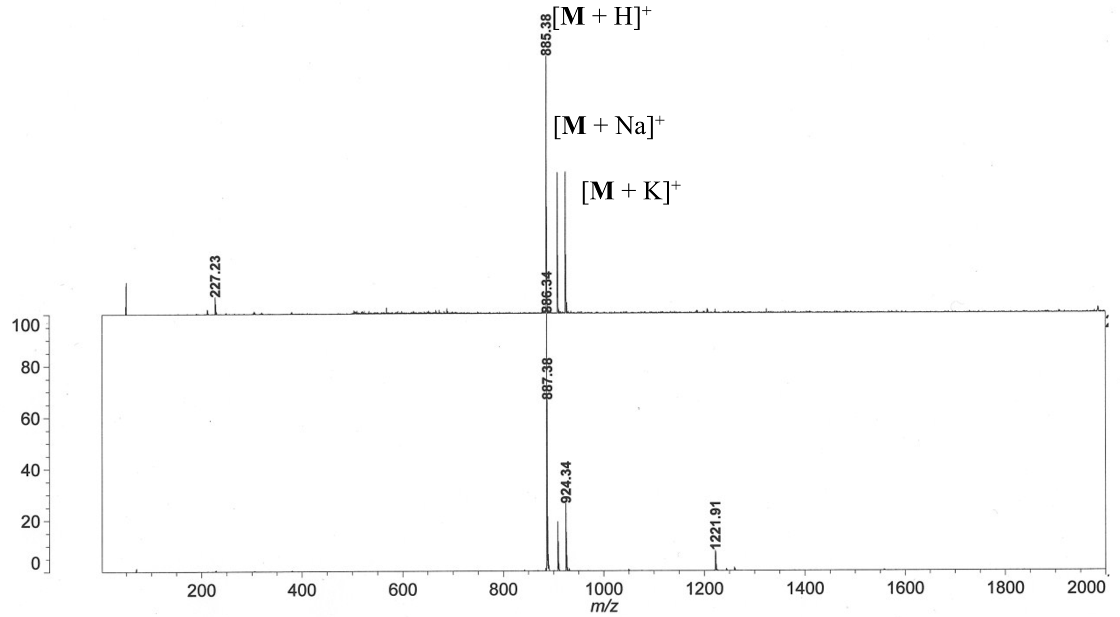

Fig. S1 MALDI-TOF mass spectra of **L-1** (lower) and **D-1** (upper). An α-CHCA was used as a matrix. **L-1**; calcd. *m*/*z* 885.50 for [M+H]$^+$, obsd. *m*/*z* 886.34, **D-1**; calcd. *m*/*z* 885.50 for [M+H]$^+$, obsd. *m*/*z* 885.38.

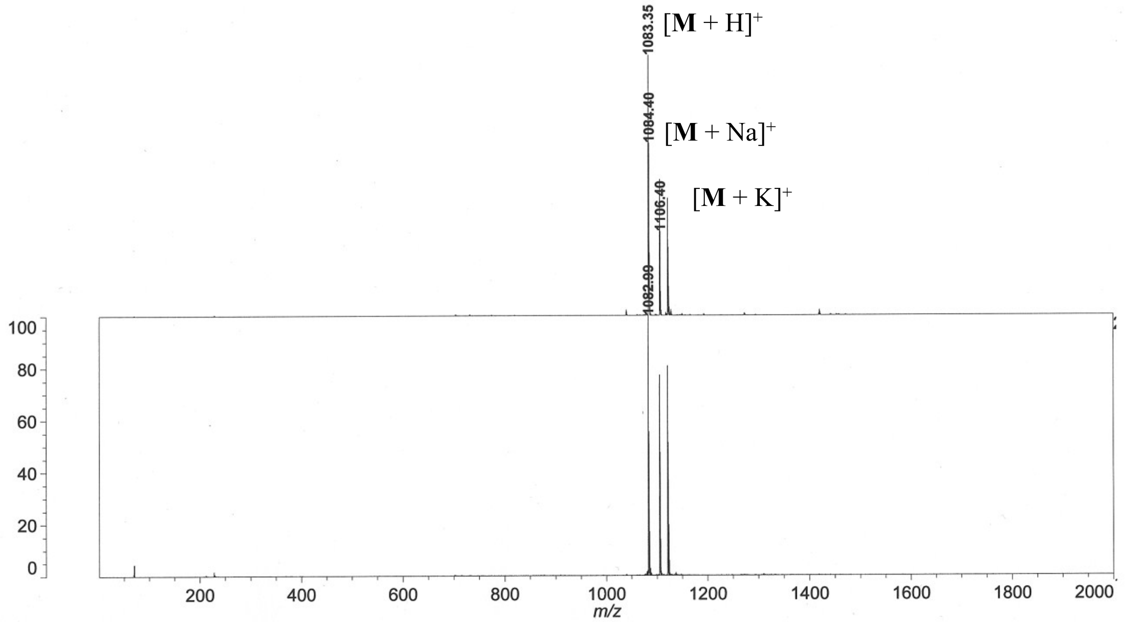

Fig. S2 MALDI-TOF mass spectra of **L-2** (lower) and **D-2** (upper). An α-CHCA was used as a matrix. **L-2**; calcd. *m*/*z* 1082.58 for [M+H]$^+$, obsd. *m*/*z* 1082.99, **D-2**; calcd. *m*/*z* 1082.58 for [M+H]$^+$, obsd. *m*/*z* 1083.55.

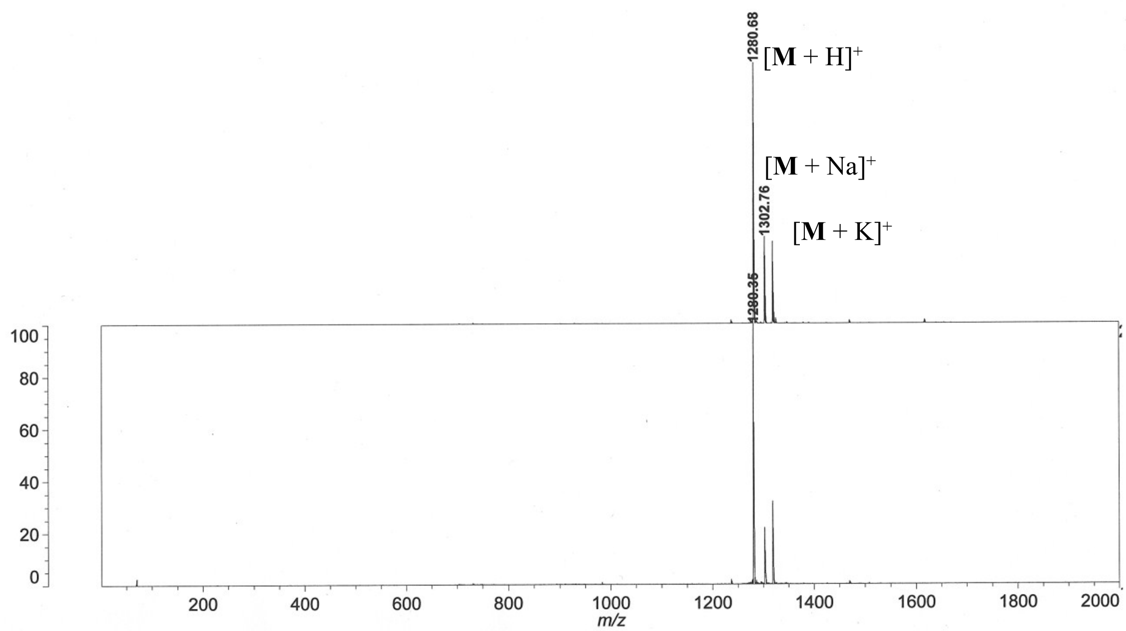

Fig. S3 MALDI-TOF mass spectra of **L-3** (lower) and **D-3** (upper). An α-CHCA was used as a matrix. **L-3**; calcd. *m*/*z* 1279.67 for $[M+H]^+$, obsd. *m*/*z* 1280.35, **D-3**; calcd. *m*/*z* 1279.67 for $[M+H]^+$, obsd. *m*/*z* 1280.68.

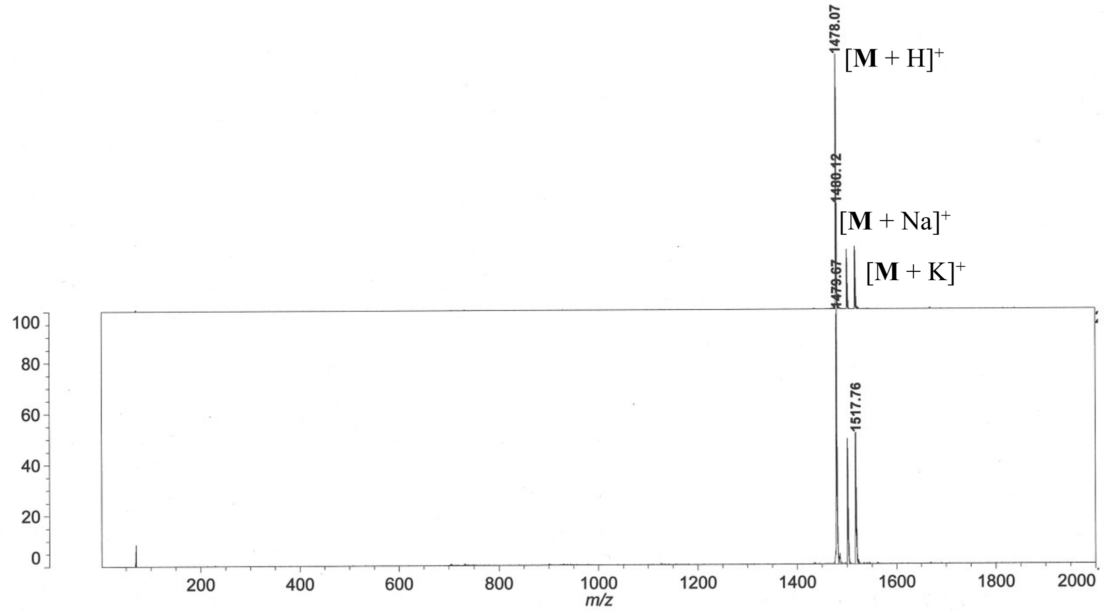

Fig. S4 MALDI-TOF mass spectra of **L-4** (lower) and **D-4** (upper). An α-CHCA was used as a matrix. **L-4**; calcd. *m*/*z* 1476.75 for $[M+H]^+$, obsd. *m*/*z* 1478.60, **D-4**; calcd. *m*/*z* 1476.75 for $[M+H]^+$, obsd. *m*/*z* 1478.07.

Measurement of absolute unpolarised photoluminescence (PL) quantum yield

The absolute unpolarised photoluminescence (PL) quantum yields were measured using an Absolute PL Quantum Yield Measurement System (C9920-02, Hamamatsu Photonics [Hamamatsu, Japan]) in air at room temperature. The excitation wavelength in $CHCl_3$ (path length 10 mm) was 300 nm. Spectroscopic-grade $CHCl_3$ (Dojindo [Kumamoto, Japan], Spectrosol) was used for spectroscopic measurements.

Measurement of photoluminescence and CPL spectra

The CPL spectra were obtained with a JASCO (Hachioji, Japan) CPL-300 spectrofluoropolarimeter at room temperature. A scattering angle of 0° was used for the excitation of non-polarised monochromatic incident light with a bandwidth of 10 nm and a bandwidth for emission of 10 nm. Scanning speed was 50 nm per min and the time constant of PMT was 8 s. The CPL and PL spectra were smoothed by 2 accumulation without any numerical smoothing. For spectroscopic measurements, spectroscopic-grade $CHCl_3$ (Dojindo [Kumamoto, Japan], Spectrosol) were used. The excitation wavelength in $CHCl_3$ solution was 300 nm (path length 10 mm).

Measurement of circular dichroism (CD) and UV-Vis absorption spectra

The circular dichroism (CD) and UV-Vis absorption spectra of the compounds in $CHCl_3$ solution states were measured using a JASCO J-820 spectropolarimeter at room temperature. The path length in solution measurement was 1 mm. Spectroscopic-grade $CHCl_3$ solutions (Dojindo [Kumamoto, Japan], Spectrosol) was used for spectroscopic measurements.

Measurement of MASS spectra

1. Mass spectrometry

Mass spectra were taken with a LCMS-IT-TOF mass spectrometer (Shimadzu Co. [Kyoto, Japan]) under the following conditions: ionization method, electrospray ionization (ESI); solvent, acetonitrile; mass range, *m/z* 100 – 4000; mode, positive; spray voltage, 4.5 kV (positive mode), or −3.5 kV (negative mode); nebulizer gas flow rate; 1.5 L min^{-1}; CDL temperature, 200 °C; heat block temperature, 200 °C; ion source pressure, 80 Pa; ion trap pressure, 1.8×10^{-2} Pa; TOF pressure, 1.4×10^{-4} Pa; resolution, >10,000. Calibration and

tuning of the instrument were carried out using an available standard solution of trifluoroacetic acid (Shimadzu Co. [Kyoto, Japan]).

Measurement of NMR spectra

^{19}F-NMR spectra were taken with a JEOL ECA 600 spectrometer. Trifluoroacetic acid in chloroform-*d* was used as the external reference (δ = −76.5 ppm).

NMR spectra were recorded on a JNM ECA-600 spectrometer (JEOL [Hachioji, Japan]). Samples were dissolved in chloroform-*d*. ^{19}F-NMR spectra were measured in the proton decoupling mode. Trifluoroacetic acid was used as an external standard (δ = −76.5 ppm in chloroform-*d*) in ^{19}F -NMR experiments.

References:

[1] N. Hara, M. Okazaki, M. Shizuma, S; Marumoto, N. Tajima, M. Fujiki and Y. Imai, *ChemistrySelect*, 2017, **2**, 10317.

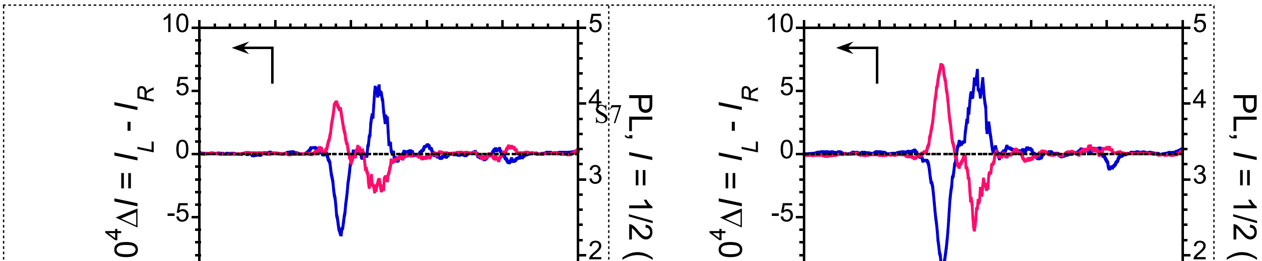

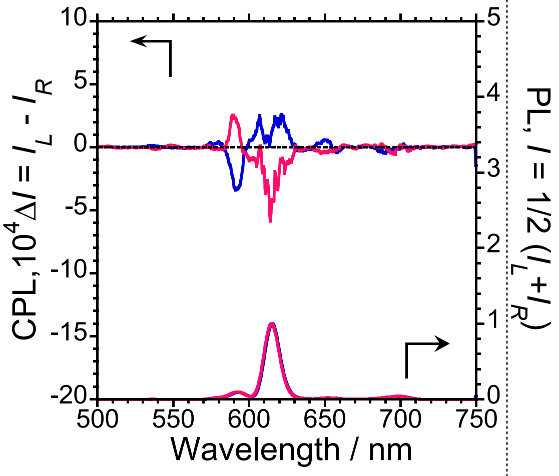

Fig. S5c L- and **D-1**/Eu(hfa)$_3$ =1/2.

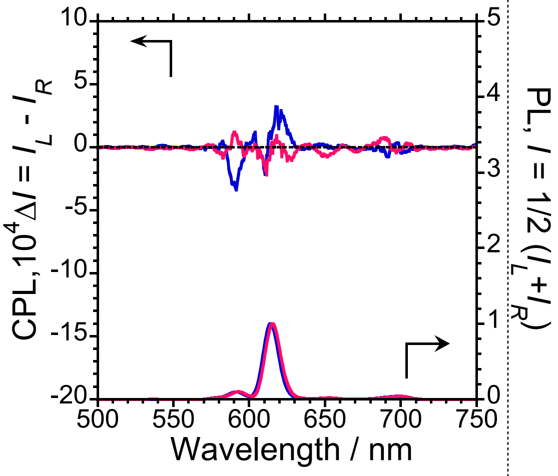

Fig. S5d L- and **D-1**/Eu(hfa)$_3$ =1/3.

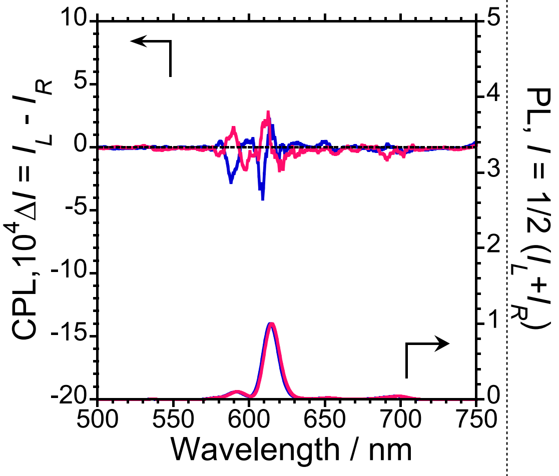

Fig. S5e L- and **D-1**/Eu(hfa)$_3$ =1/4.

Fig. S5 CPL and PL spectra as a function of molar ratio of **L-1** (blue lines) (and **D-1** (red lines)) to Eu(hfa)$_3$ in EtOH-free chloroform. Path length 10 mm. λ_{ex} = 300 nm. [Eu(hfa)$_3$]$_0$ = 1.0 × 10^{-4} M. Bandwidth for emission 10 nm, bandwidth for excitation 10 nm, response time of PMT 8 s, scanning rate 50 nm per min, and two-time scan.

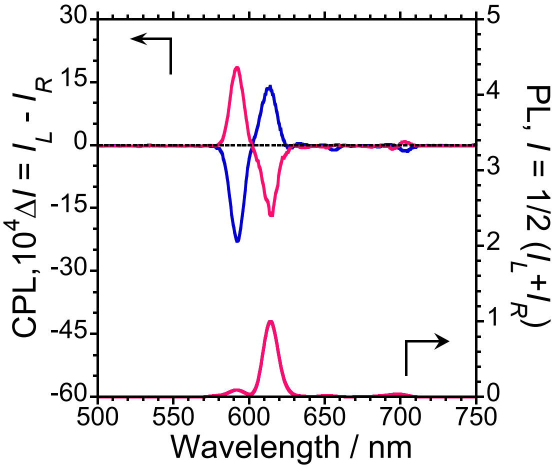

Fig. S6a L- and **D-2**/Eu(hfa)$_3$ =1/0.5.

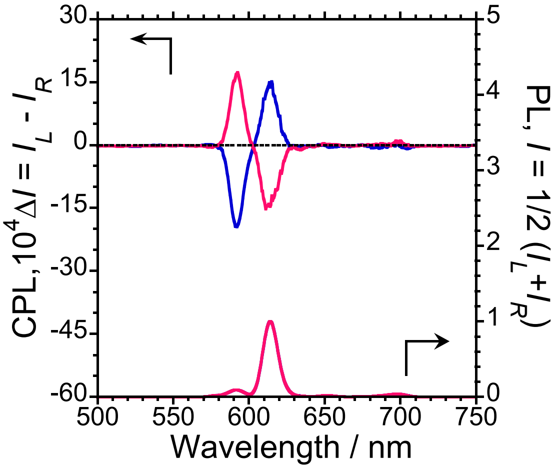

Fig. S6b L- and **D-2**/Eu(hfa)$_3$ =1/1.

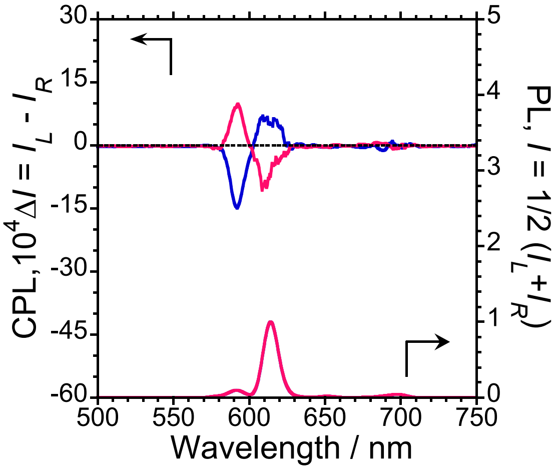

Fig. S6c **L**- and **D-2**/Eu(hfa)$_3$ =1/2.

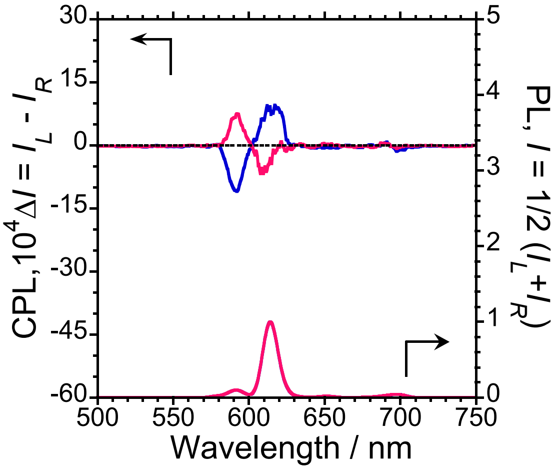

Fig. S6d **L**- and **D-2**/Eu(hfa)$_3$ =1/3.

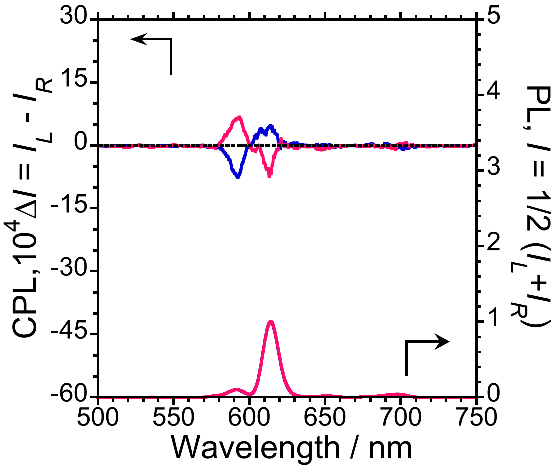

Fig. S6e **L**- and **D-2**/Eu(hfa)$_3$ =1/4.

Fig. S6 CPL and PL spectra as a function of molar ratio of **L-2** (blue lines) (and **D-2** (red lines)) to Eu(hfa)$_3$ in EtOH-free chloroform. Path length 10 mm. λ_{ex} = 300 nm. [Eu(hfa)$_3$]$_0$ = 1.0 × 10^{-4} M. Bandwidth for emission 10 nm, bandwidth for excitation 10 nm, response time of PMT 8 s, scanning rate 50 nm per min, and two-time scan.

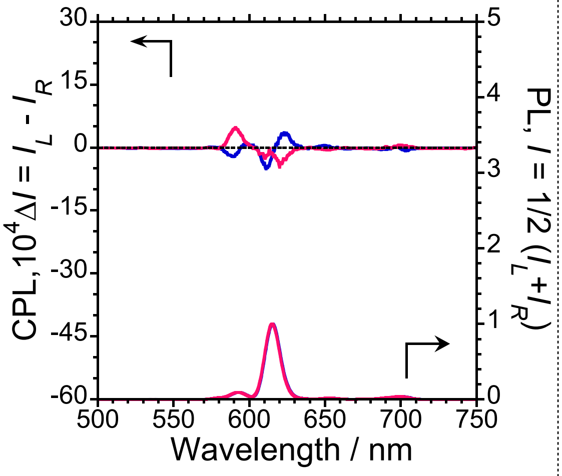

Fig. S7a **L**- and **D-3**/Eu(hfa)$_3$ =1/0.5.

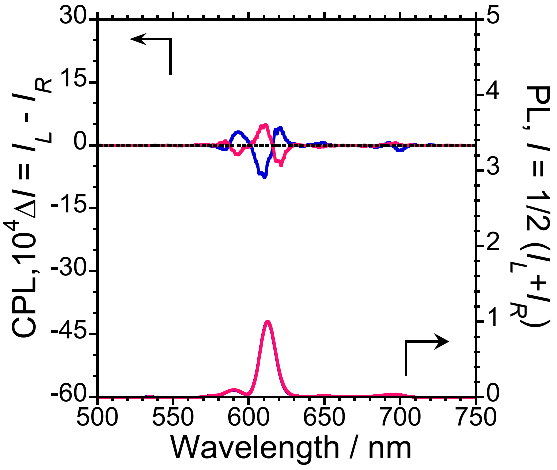

Fig. S7b **L**- and **D-3**/Eu(hfa)$_3$ =1/1.

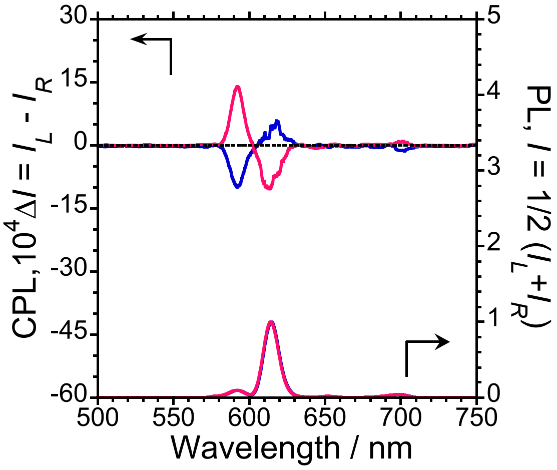

Fig. S7c L- and **D-3**/Eu(hfa)$_3$ =1/2.

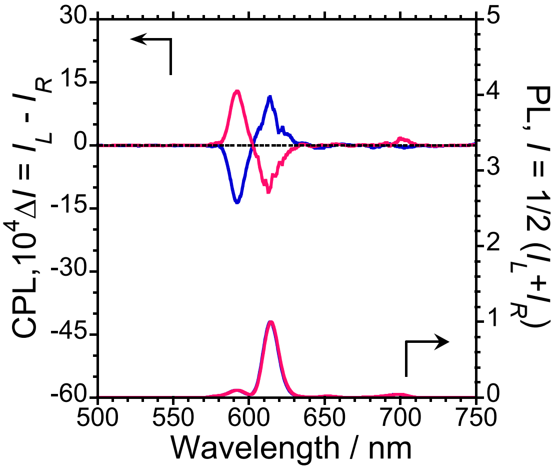

Fig. S7d L- and **D-3**/Eu(hfa)$_3$ =1/3.

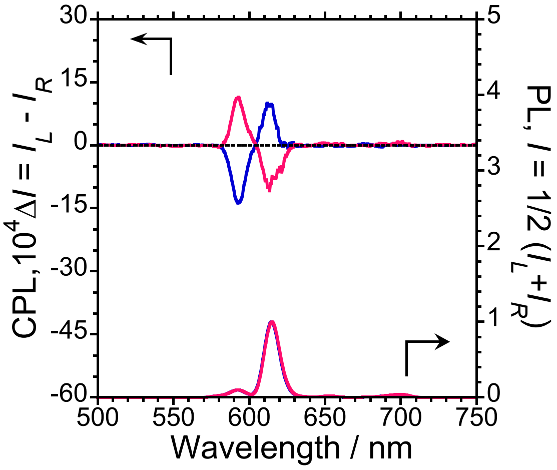

Fig. S7e L- and **D-3**/Eu(hfa)$_3$ =1/4.

Fig. S7 CPL and PL spectra as a function of molar ratio of **L-3** (blue lines) (and **D-3** (red lines)) to Eu(hfa)$_3$ in EtOH-free chloroform. Path length 10 mm. λ_{ex} = 300 nm. [Eu(hfa)$_3$]$_0$ = 1.0×10^{-4} M. Bandwidth for emission 10 nm, bandwidth for excitation 10 nm, response time of PMT 8 s, scanning rate 50 nm per min, and two-time scan.

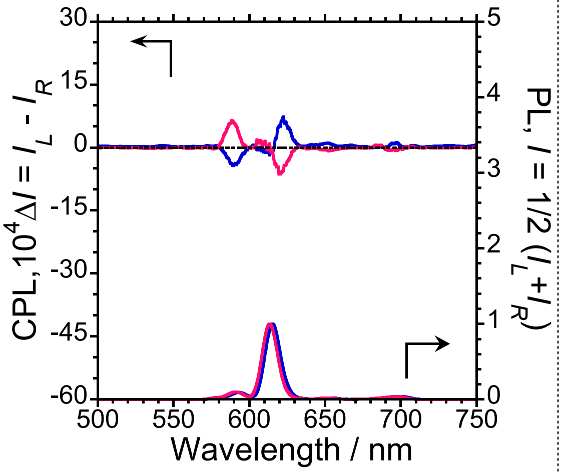

Fig. S8a L- and **D-4**/Eu(hfa)$_3$ =1/0.5.

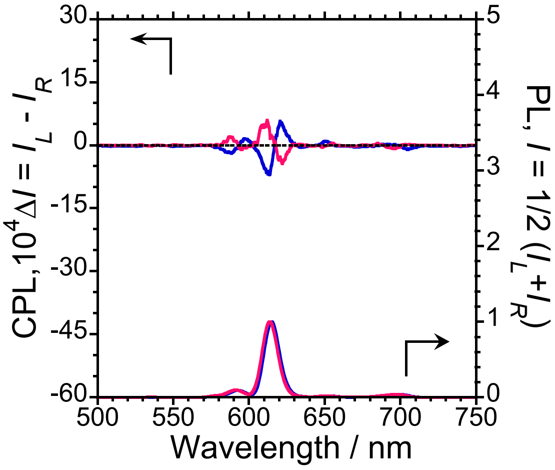

Fig. S8b L- and **D-4**/Eu(hfa)$_3$ =1/1.

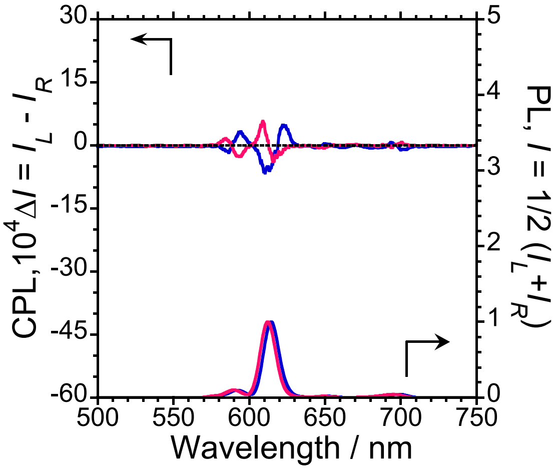

Fig. S8c **L**- and **D-4**/Eu(hfa)$_3$ =1/2.

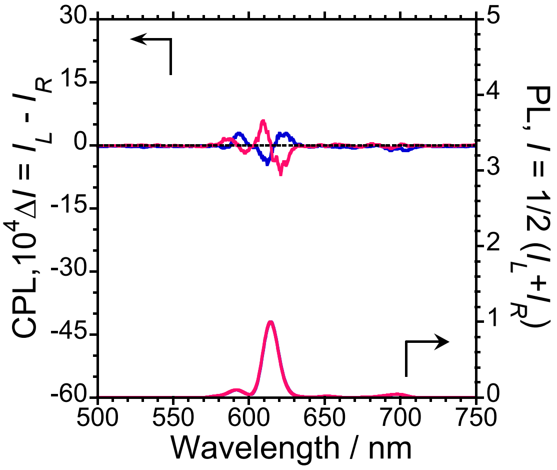

Fig. S8d **L**- and **D-4**/Eu(hfa)$_3$ =1/3.

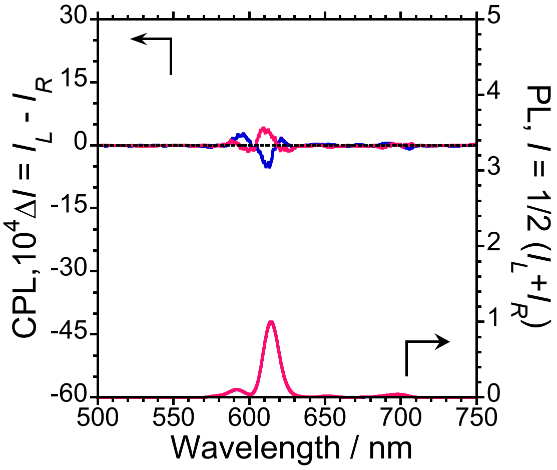

Fig. S8e **L**- and **D-4**/Eu(hfa)$_3$ =1/4.

Fig. S8 CPL and PL spectra as a function of molar ratio of **L-4** (blue lines) (and **D-4** (red lines)) to Eu(hfa)$_3$ in EtOH-free chloroform. Path length 10 mm. λ_{ex} = 300 nm. [Eu(hfa)$_3$]$_0$ = 1.0 × 10^{-4} M. Bandwidth for emission 10 nm, bandwidth for excitation 10 nm, response time of PMT 8 s, scanning rate 50 nm per min, and two-time scan.

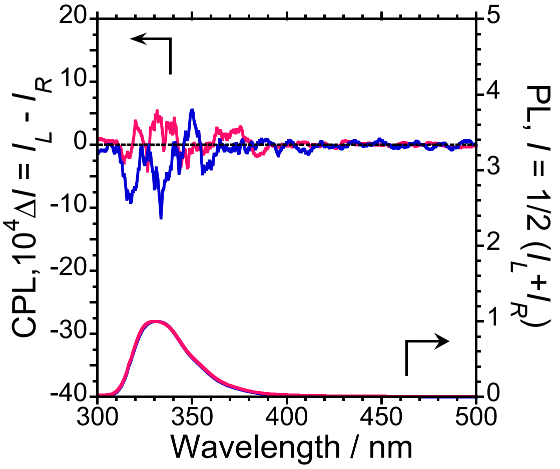

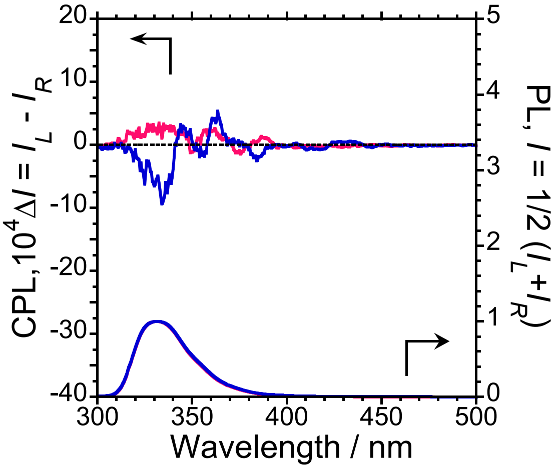

**Fig. S9a L**- and **D-1**.	**Fig. S9b L**- and **D-2**.

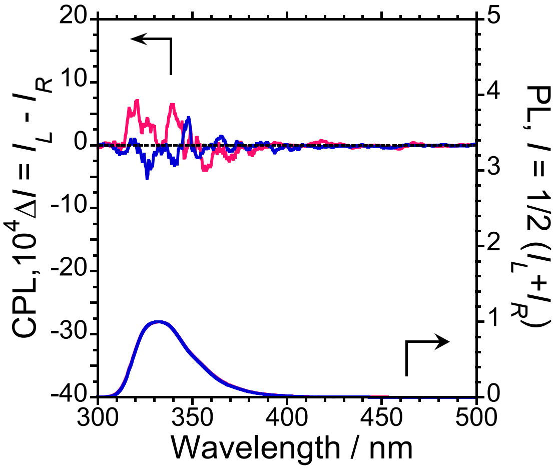

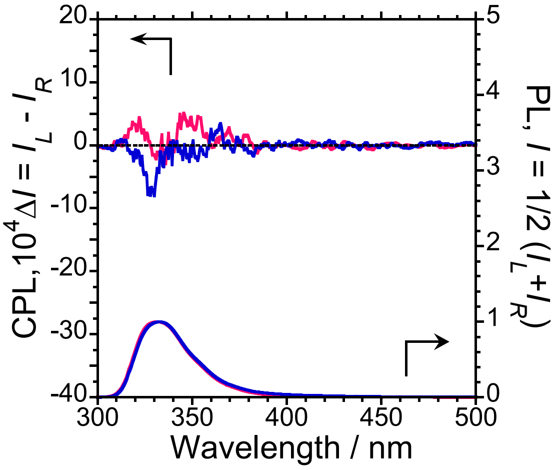

**Fig. S9c L**- and **D-3**.	**Fig. S9d L**- and **D-4**.

Fig. S9 CPL and PL spectra of (a) **1**, (b) **2**, (c) **3**, and (d) **4** in EtOH-free chloroform. Blue and red lines are **L**- and **D**-isomers, respectively. Path length 10 mm. λ_{ex} = 270 nm. Conc. = 1.0 × 10^{-4} M. Bandwidth for emission 10 nm, bandwidth for excitation 10 nm, response time of PMT 8 s, scanning rate 50 nm per min, and two-time scan.

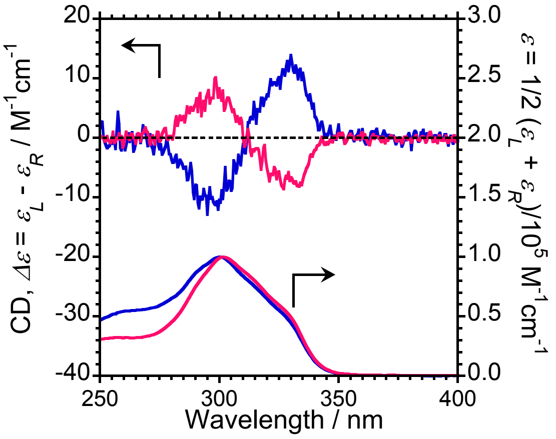

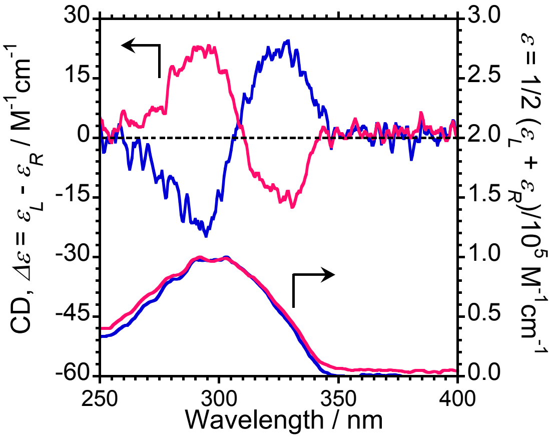

Fig. S10a L- and **D-1**/Eu(hfa)$_3$ =1/1. **Fig. S10b L-** and **D-2**/Eu(hfa)$_3$ =1/1.

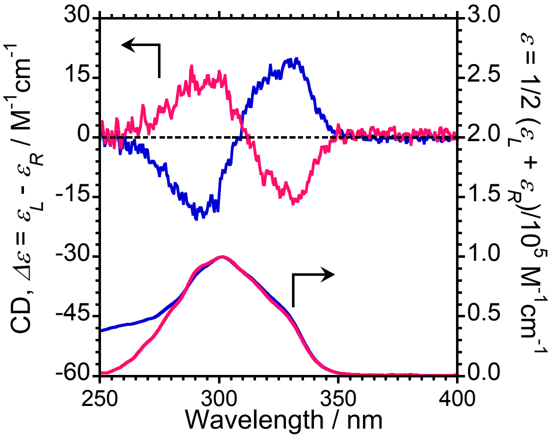

Fig. S10c L- and **D-3**/Eu(hfa)$_3$ =1/1.

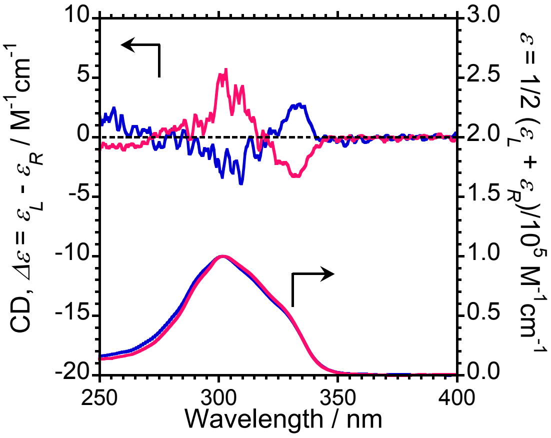

Fig. S10d L- and **D-4**/Eu(hfa)$_3$ =1/1.

Fig. S10 Comparison of CD and UV spectra of (a) **1**/Eu(hfa)$_3$ = 1/1, (b) **2**/Eu(hfa)$_3$ = 1/1, (c) **3**/Eu(hfa)$_3$ = 1/1, and (d) **4**/Eu(hfa)$_3$ = 1/1 (in nominal molar ratio) in EtOH-free chloroform. Blue and red lines are **L-** and **D-**isomers, respectively. Path lengths were commonly 1 mm. $[Eu]_0$= 1.0 × 10^{-4} M in EtOH-free chloroform.

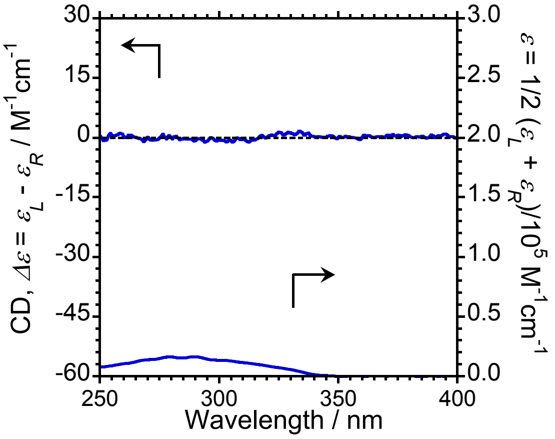

Fig. S11a L-3/Eu(hfa)$_3$ =1/0.5.

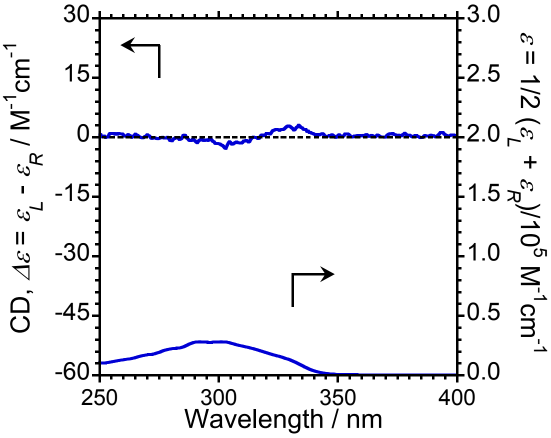

Fig. S11b L-3/Eu(hfa)$_3$ =1/1.

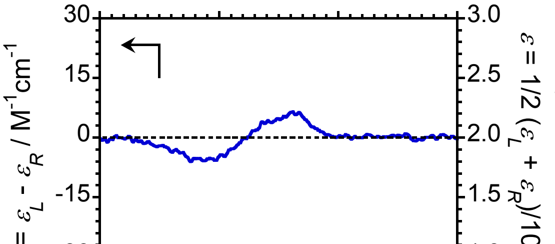

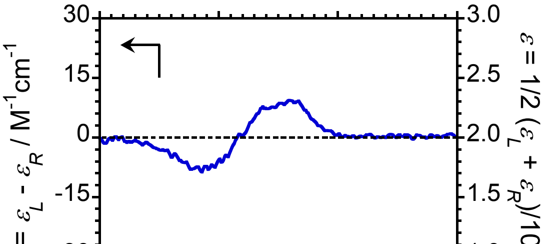

Fig. S11 Comparison of CD and UV spectra of (a) **L-3**/Eu(hfa)$_3$ = 1/0.5, (b) **L-3**/Eu(hfa)$_3$ = 1/1, (c) **L-3**/Eu(hfa)$_3$ = 1/2, and **L-3**/Eu(hfa)$_3$ = 1/3 (in nominal molar ratio) in EtOH-free chloroform. Blue lines are **L**-isomers, respectively. Path lengths were commonly 1 mm. $[Eu]_0$= 1.0 × 10^{-4} M in EtOH-free chloroform.

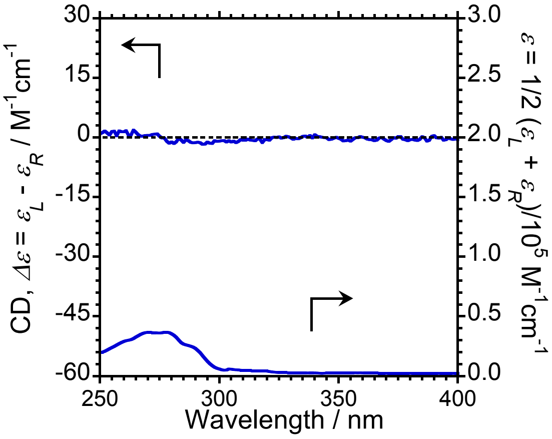

Fig. S12a L-1.

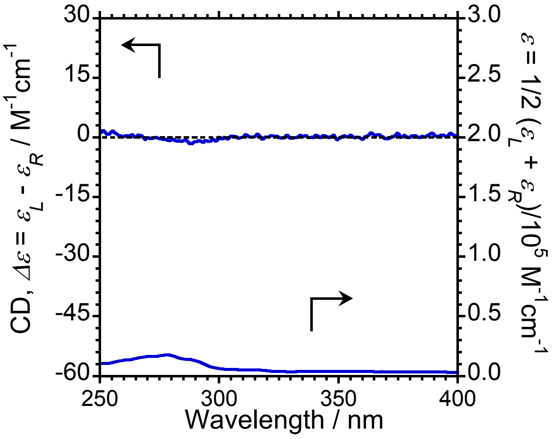

Fig. S12b L-2.

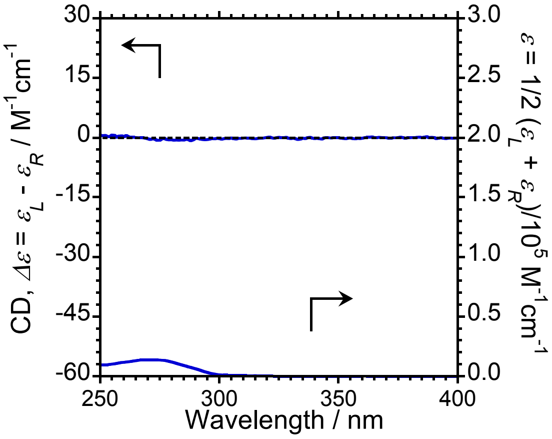

Fig. S12c L-3.

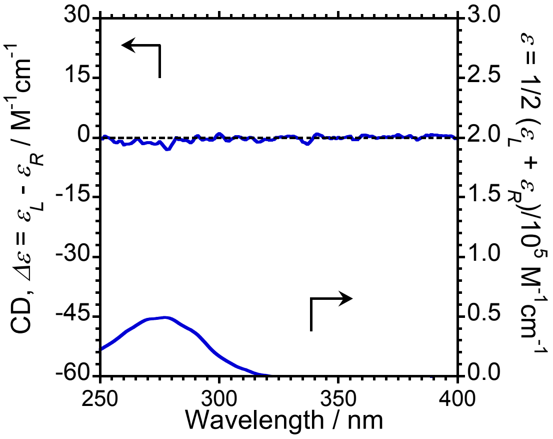

Fig. S12d L-4.

Fig. S12 Comparison of CD and UV spectra of (a) **L-1**, (b) **L-2**, (c) **L-3**, and (d) **L-4** in EtOH-free chloroform. Conc. = 1.0 × 10^{-4} M. Path lengths were 1 mm. (Path length of only **1**

was 10 mm.)

(a)

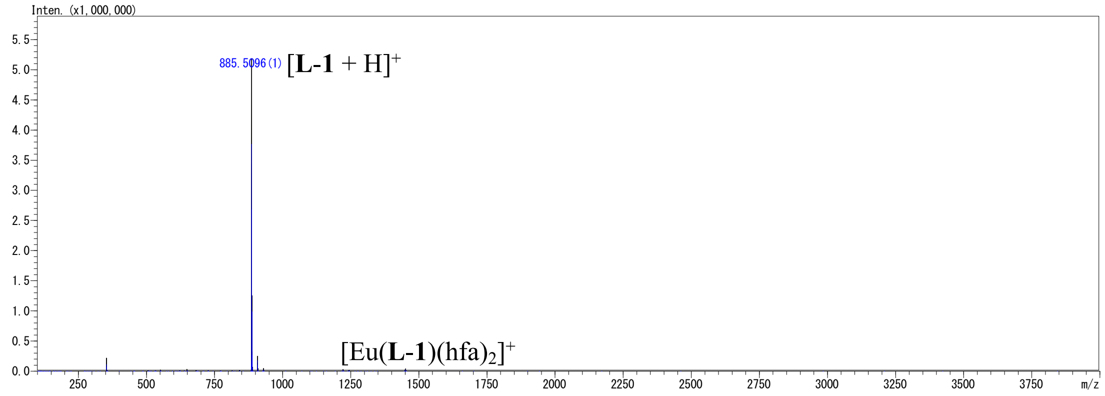

(b)

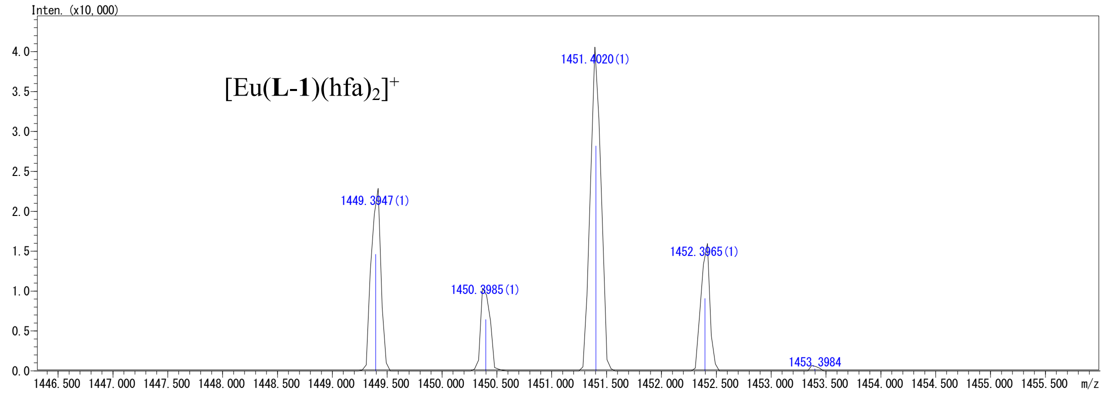

Fig. S13 Mass spectra of **L-1**/Eu(hfa)$_3$ (1/1)

(a) All range, and (b) expanded range. Found *m/z* 1451.4020, calcd. *m/z* 1451.3970 as [Eu(**L-1**)(hfa)$_2$]$^+$.

(a)

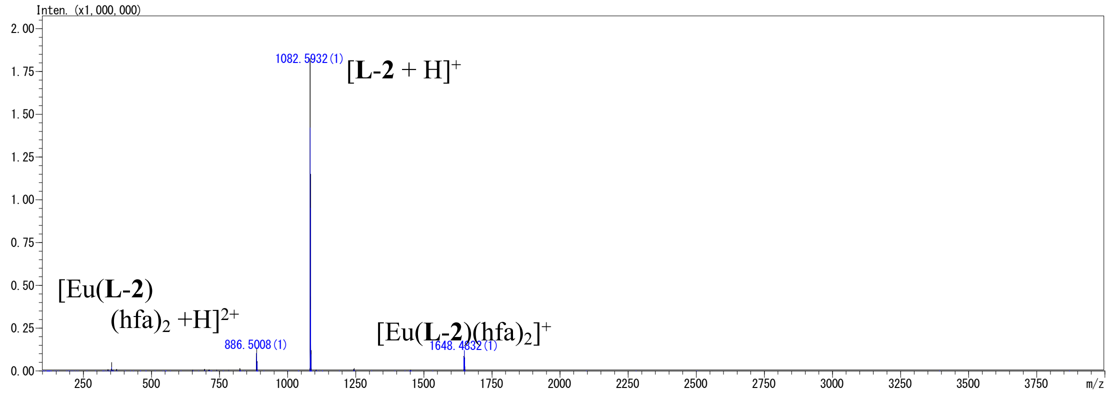

(b)

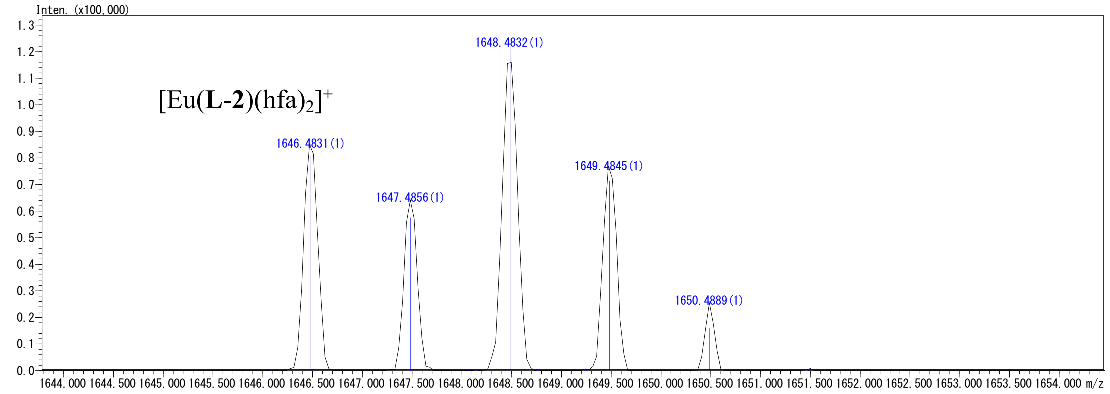

Fig. S14 Mass spectra of **L-2**/Eu(hfa)$_3$ (1/1)

(a) All range, and (b) expanded range. Found *m/z* 1648.4832, calcd. *m/z* 1648.4814 as [Eu(**L-2**)(hfa)$_2$]$^+$.

(a)

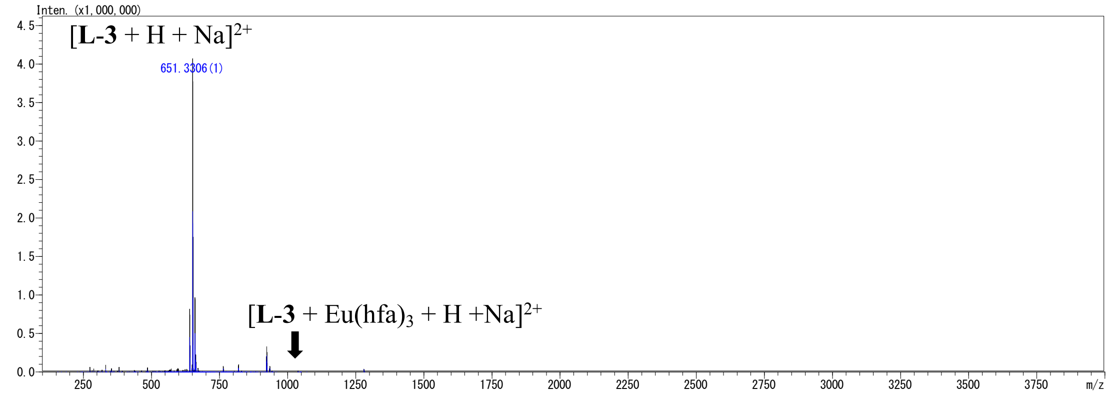

(b)

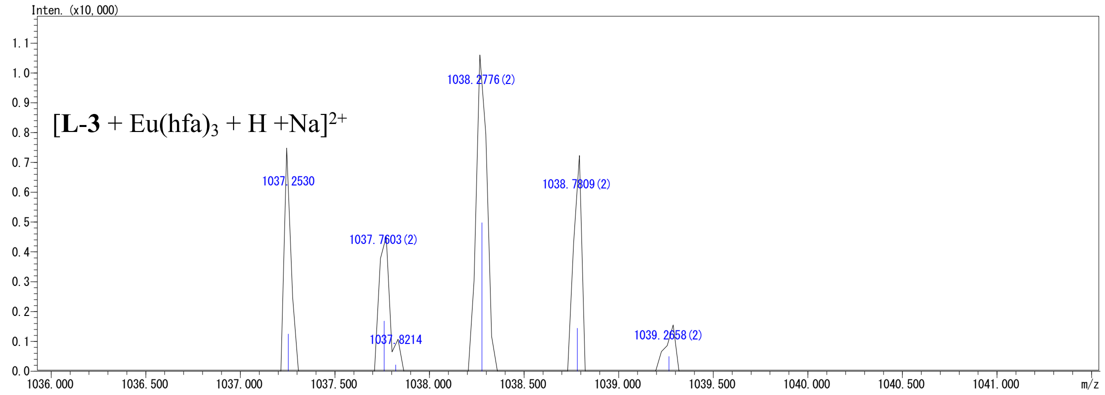

Fig. S15 Mass spectra of **L-3**/Eu(hfa)$_3$ (1/1)

(a) All range, and (b) expanded range. Found *m/z* 1037.2530, calcd. *m/z* 1037.2743 as [Eu(**L-3**)(hfa)$_3$ + H +Na]$^{2+}$.

(a)

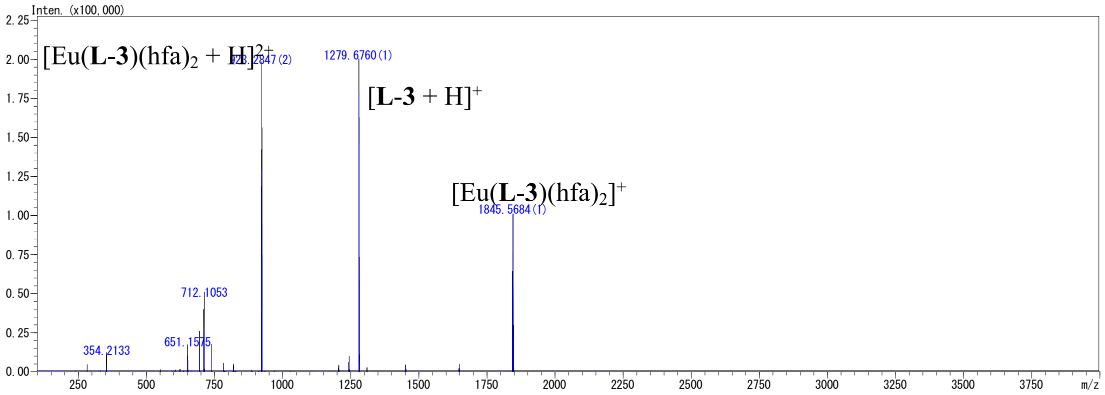

(b)

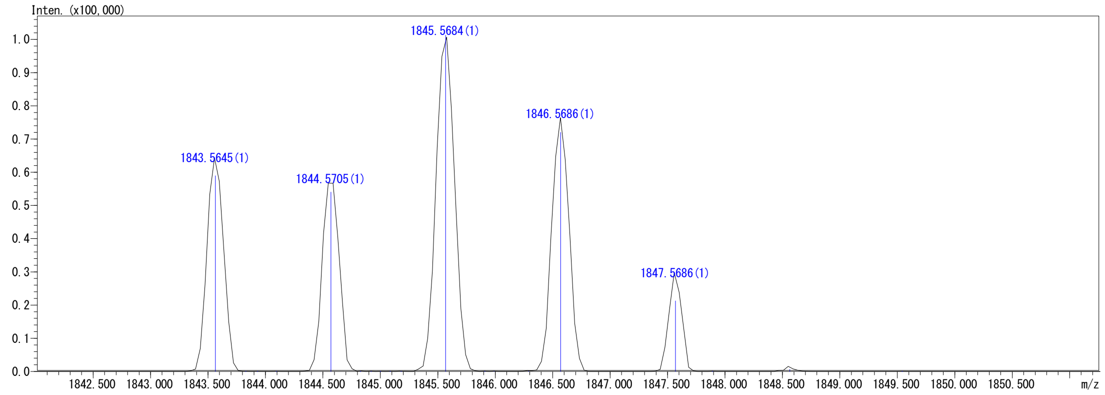

Fig. S16 Mass spectra of **L-3**/Eu(hfa)$_3$ (1/2)

(a) All range, and (b) expanded range. Found *m/z* 1845.5684, calcd. *m/z* 1845.5657 as [Eu(**L-3**)(hfa)$_2$]$^{+}$

(a)

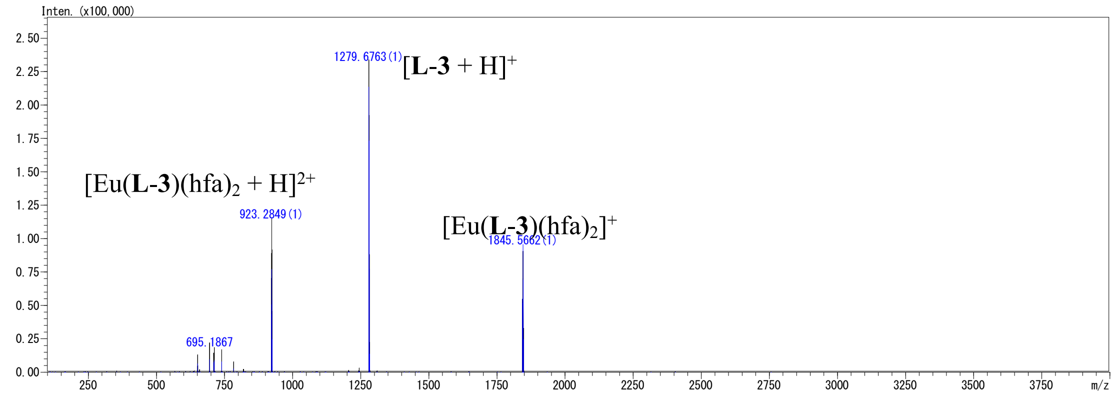

(b)

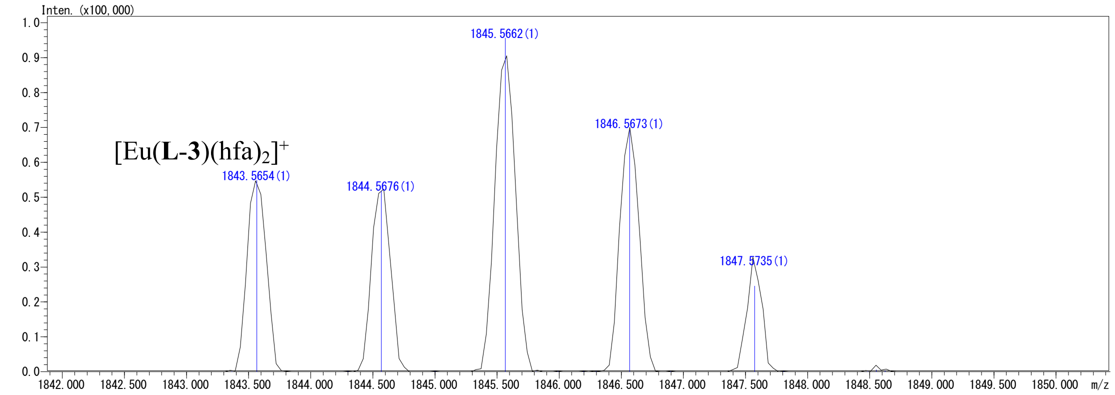

Fig. S17 Mass spectra of **L-3**/Eu(hfa)$_3$ (1/3)

(a) All range, and (b) expanded range. Found *m/z* 1845.5662, calcd. *m/z* 1845.5657 as [Eu(**L-3**)(hfa)$_2$]$^+$.

(a)

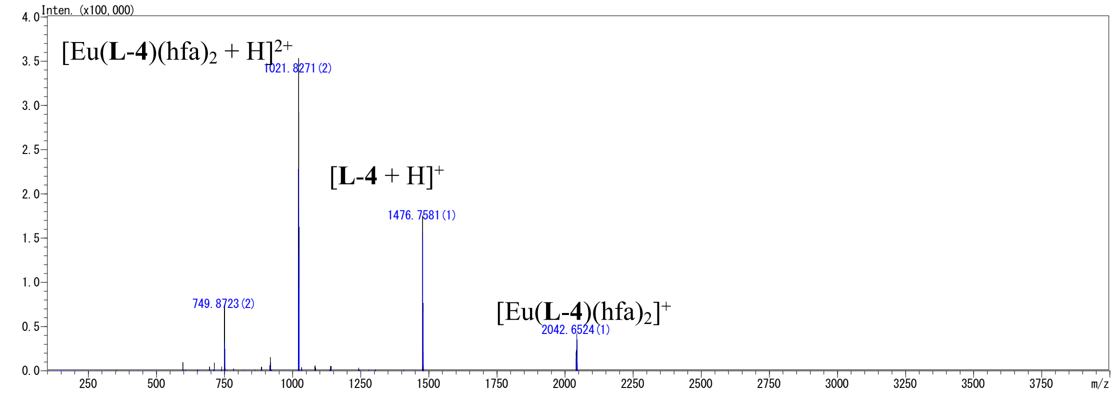

(b)

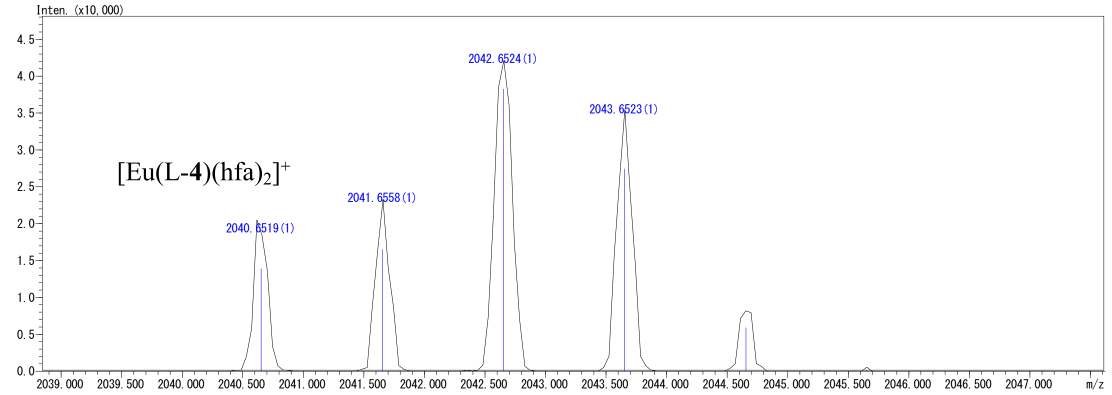

Fig. S18 Mass spectra of **L-4**/Eu(hfa)$_3$ (1/1)

(a) All range, and (b) expanded range. Found *m/z* 2042.6524, calcd. *m/z* 2042.6501 as [Eu(**L-4**)(hfa)$_2$]$^+$.

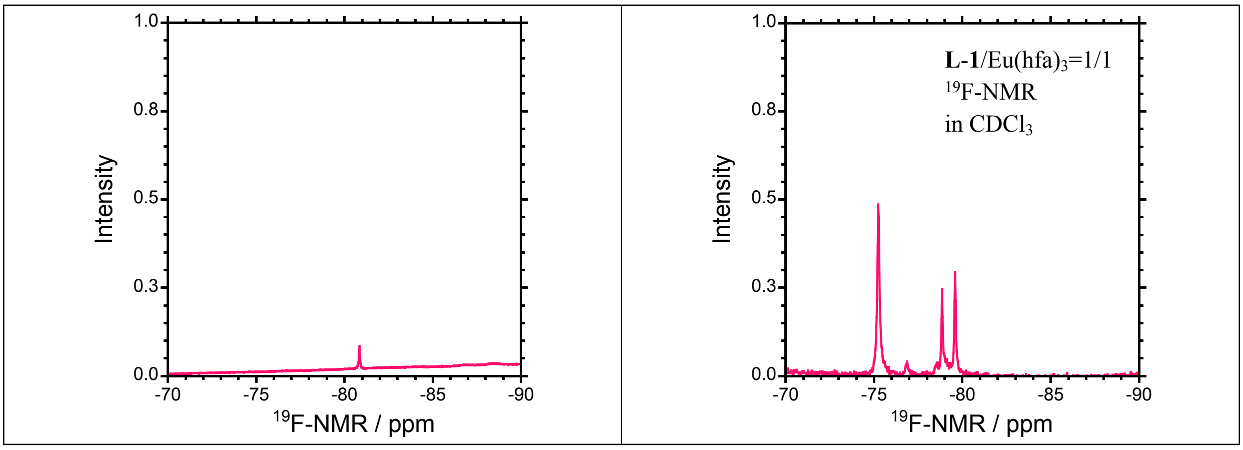

Fig. S19 ^{19}F-NMR spectrum of Eu(hfa)$_3$ and **L-1**/Eu(hfa)$_3$ (1/1) in CDCl$_3$

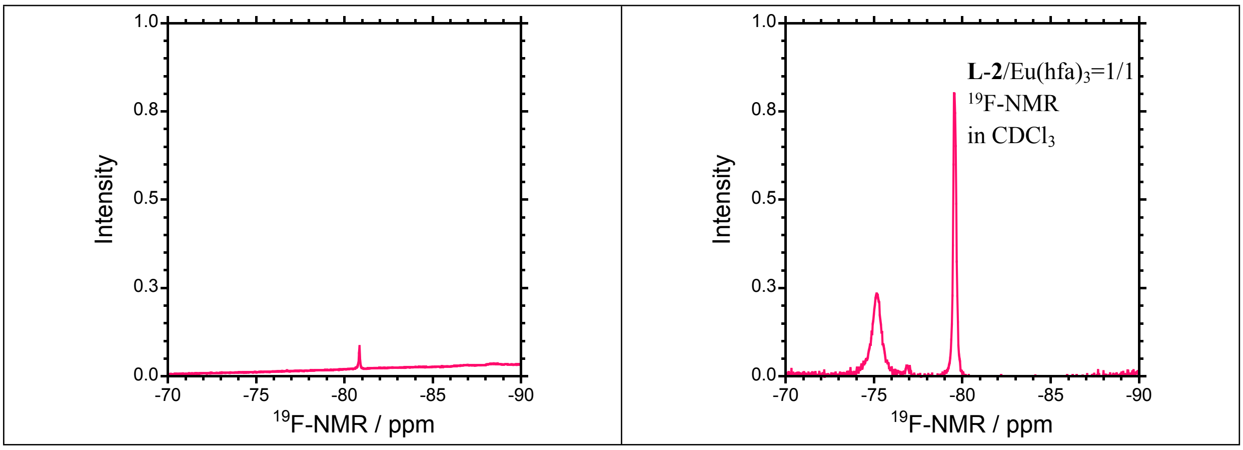

Fig. S20 ^{19}F-NMR spectrum of Eu(hfa)$_3$ and **L-2**/Eu(hfa)$_3$ (1/1) in CDCl$_3$

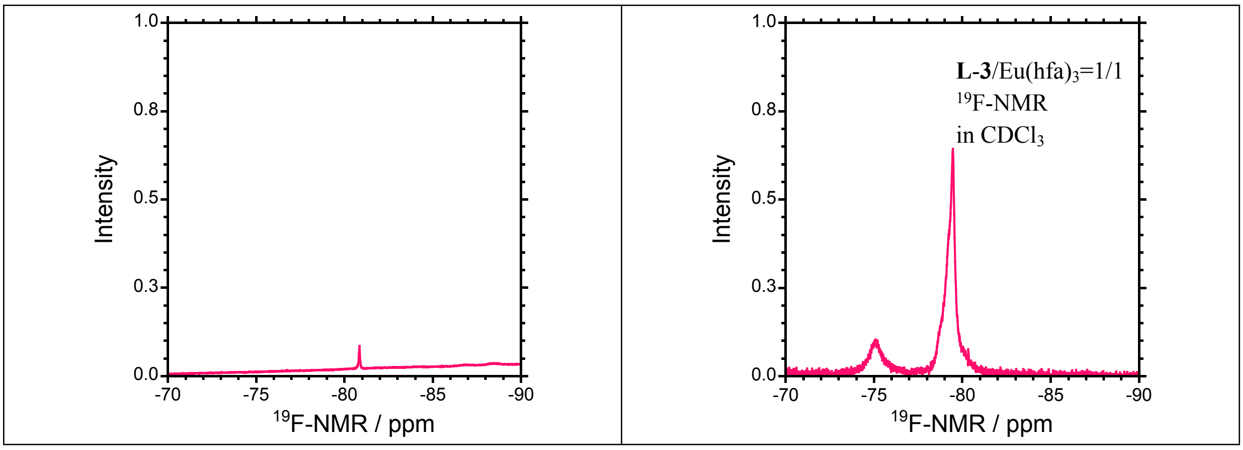

Fig. S21 ^{19}F-NMR spectrum of Eu(hfa)$_3$ and **L-3**/Eu(hfa)$_3$ (1/1) in $CDCl_3$

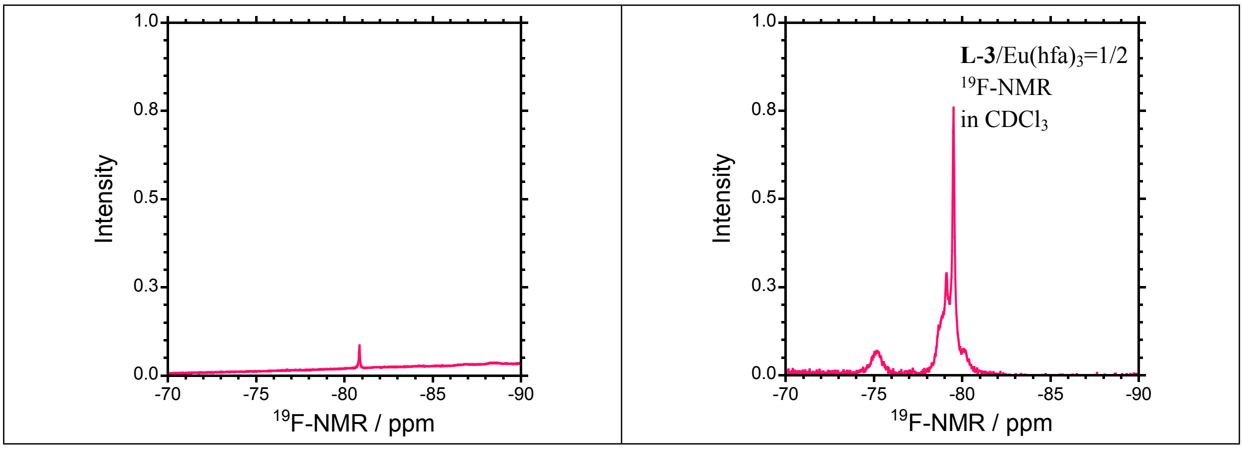

Fig. S22 ^{19}F-NMR spectrum of Eu(hfa)$_3$ and **L-3**/Eu(hfa)$_3$ (1/2) in $CDCl_3$

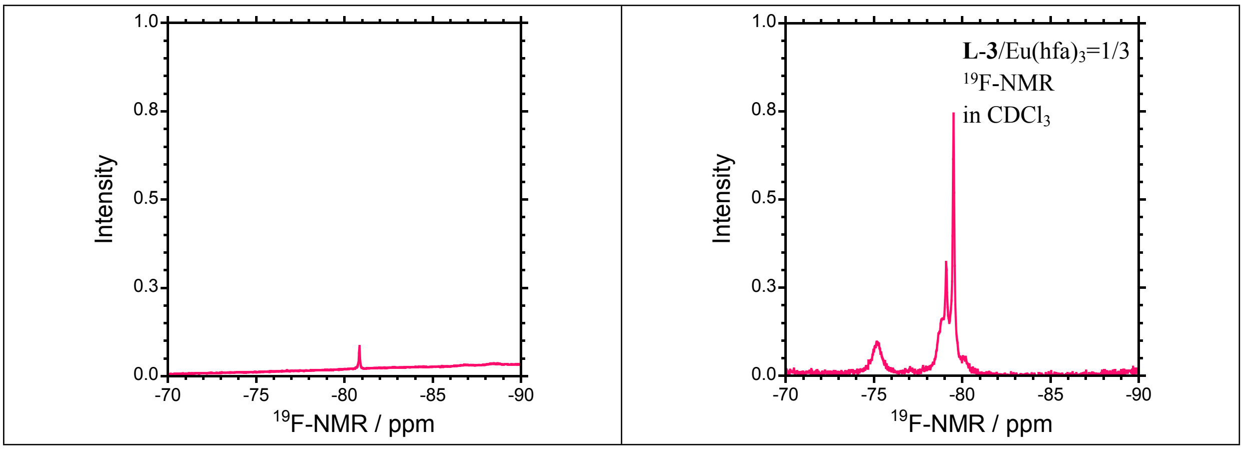

Fig. S23 ^{19}F-NMR spectrum of Eu(hfa)$_3$ and **L-3**/Eu(hfa)$_3$ (1/3) in $CDCl_3$

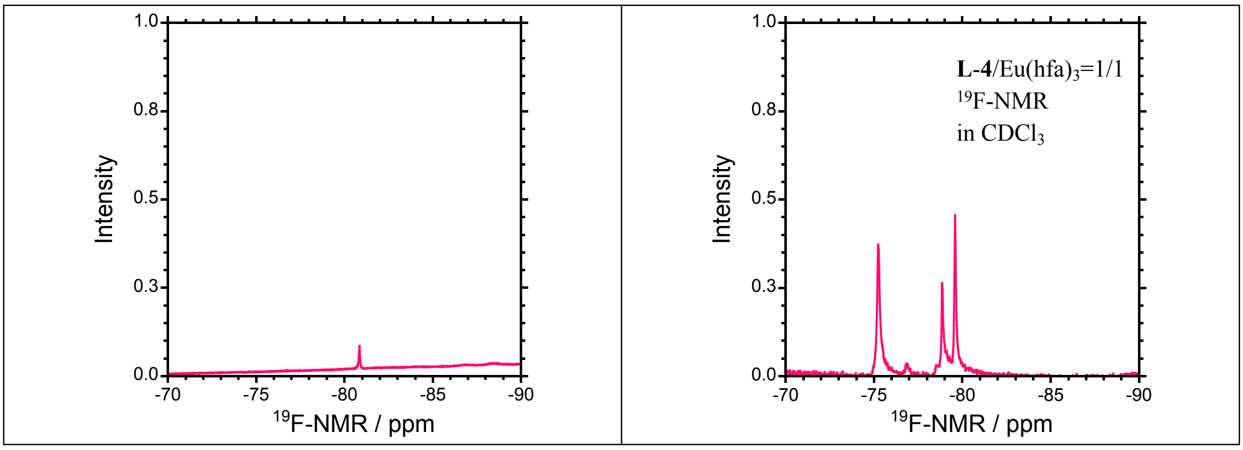

Fig. S24 ^{19}F-NMR spectrum of Eu(hfa)$_3$ and **L-4**/Eu(hfa)$_3$ (1/1) in $CDCl_3$
